# Redox Chemistries for Vacancy Modulation in Plasmonic Copper Phosphide Nanocrystals

**DOI:** 10.1021/acsnano.3c08962

**Published:** 2024-02-07

**Authors:** Alexander G. Rachkov, Kevin Chalek, Hang Yin, Mingjie Xu, Gregory P. Holland, Alina M. Schimpf

**Affiliations:** † Department of Chemistry and Biochemistry, University of California, San Diego, La Jolla, California 92093, United States; ‡ Department of Chemistry and Biochemistry, San Diego State University, San Diego, California 92182, United States; § Irvine Materials Research Institute (IMRI) University of California, Irvine, California 92697, United States; ∥ Program in Materials Science and Engineering, University of California, San Diego, La Jolla, California 92093, United States

**Keywords:** copper phosphide, nanocrystals, electronic doping, defect doping, localized surface planson resonance

## Abstract

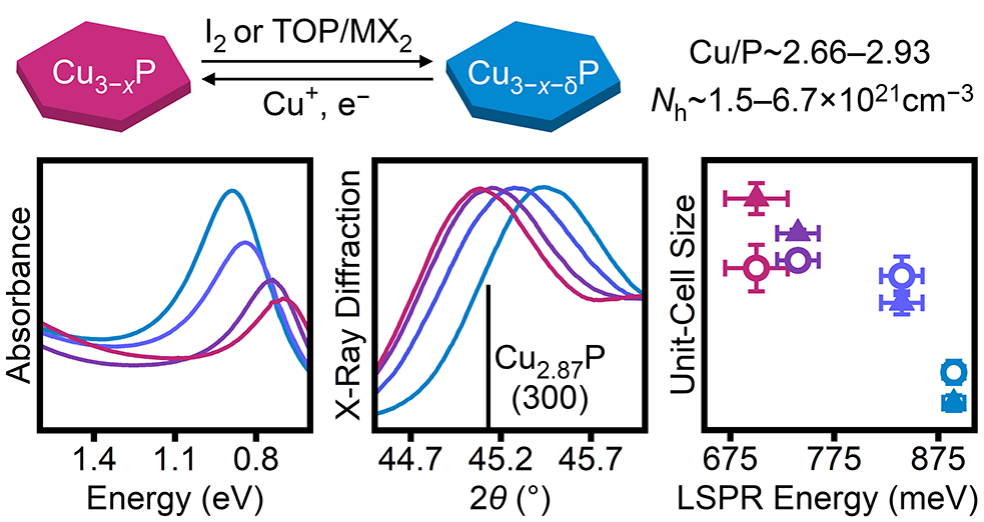

Copper phosphide (Cu_3–*x*_P) nanocrystals are promising materials for nanoplasmonics due to their substoichiometric composition, enabling the generation and stabilization of excess delocalized holes and leading to localized surface plasmon resonance (LSPR) absorption in the near-IR. We present three Cu-coupled redox chemistries that allow postsynthetic modulation of the delocalized hole concentrations and corresponding LSPR absorption in colloidal Cu_3–*x*_P nanocrystals. Changes in the structural, optical, and compositional properties are evaluated by powder X-ray diffraction, electronic absorption spectroscopy, ^31^P magic-angle spinning solid-state nuclear magnetic resonance spectroscopy, and elemental analysis. The redox chemistries presented herein can be used to access nanocrystals with LSPR energies of 660–890 meV, a larger range than has been possible through synthetic tuning alone. In addition to utilizing previously reported redox chemistries used for copper chalcogenide nanocrystals, we show that the largest structural and LSPR modulation is achieved using a divalent metal halide and trioctylphosphine. Specifically, nanocrystals treated with zinc iodide and trioctylphosphine have the smallest unit-cell volume (295.2 Å^3^) reported for *P*6_3_*cm* Cu_3–*x*_P, indicating more Cu vacancies than have been previously observed. Overall, these redox chemistries present valuable insight into controlling the optical and structural properties of Cu_3–*x*_P.

## Introduction

Cu_3–*x*_P has drawn increased attention in recent years for use in an array of applications including lithium-ion batteries,^1,2^ catalysis,^3,4^ photodetection,^5^ supercapacitors,^6^ chemodynamic therapy,^7^ and cation exchange^8,9^ and as a precursor to ternary copper chalcophosphates used in photovoltaics.^10,11^ The intrinsic nonstoichiometry arising from copper vacancies in Cu_3–*x*_P has been computationally predicted to span a range of 0.17 < *x* < 0.33,^8^ consistent with compositions experimentally validated by elemental analysis.^12,13^ The copper vacancies are charge-compensated by excess delocalized holes, which, at the nanoscale, yield an emergent localized surface plasmon resonance (LSPR) absorption in the near-IR.^8,14−17^ Despite this intrinsically hole-doped nature, strategies for dynamic redox tuning of the carrier density that have been widely demonstrated in other colloidal semiconductor nanocrystal systems^18−32^ have not yet been applied to Cu_3–*x*_P.

Electronically doped colloidal semiconductor nanocrystals^18,33−37^ typically exhibit charge-carrier modulation upon postsynthetic redox or photoredox manipulation,^18,27,33,38,39^ or via synthetically controlled defect incorporation and activation.^18−26^ Carrier-density-tuning, most typically achieved through postsynthetic redox treatments, has been demonstrated for copper chalcogenides,^21,25,28,29,31,40−44^ which also support delocalized holes to compensate copper vacancies. Due to the tunable LSPR energy and available surface chemistries, copper chalcogenide nanocrystals have shown the potential to complement or outperform noble metal nanoparticles in applications including surface enhanced Raman spectroscopy,^45^ plasmon enhanced chemical conversion,^46^ upconversion enhancement,^47^ and phototherapeutics.^48^ In contrast to the copper chalcogenides, which have shown phase-transformations with dynamic redox tuning,^49^ Cu_3–*x*_P has only one Cu-rich phase at ambient conditions. This phase-stability is analogous to that of noble metal nanoparticles, but the distinct surface chemistry, carrier properties, and potential for redox tuning are more analogous to electronically doped semiconducting copper chalcogenides. This combination makes Cu_3–*x*_P a nonredundant expansion of the available library of plasmonic nanomaterials. The ability to modulate Cu_3–*x*_P carrier density may allow its extension to nanoplasmonic applications based on plasmon-enhanced chemical conversion,^46^ ultrafast optical switching,^50,51^ and surface-enhanced Raman scattering^45^ which have already been demonstrated for an analogous class of materials, copper chalcogenide (CuE or Cu_2–*x*_E; E = S, Se, Te) nanocrystals.

Here, we develop three anaerobic, postsynthetic, redox chemistries for colloidal Cu_3–*x*_P nanocrystals and characterize the corresponding changes to the optical and structural properties using electronic absorption spectroscopy, powder X-ray diffraction, solid-state ^31^P magic-angle spinning (MAS) nuclear magnetic resonance (NMR) spectroscopy and elemental analysis. Two of the postsynthetic chemistries, reduction with Cu^+^ and oxidation with I_2_, have been applied to obtain similar Cu-coupled redox modulation in copper chalcogenide nanocrystals.^28,31,40,52−54^ The third treatment, which utilizes a divalent metal halide and trioctylphosphine (TOP) is shown to yield the greatest structural and optical changes. The postsynthetic chemistries and characterizations detailed in this study provide the groundwork for broad tunability of the composition and associated carrier density and optical properties of Cu_3–*x*_P nanocrystals.

## Results and Analysis

Cu_3–*x*_P nanocrystals were synthesized via a previously developed one-pot, heat-up reaction.^16^ In a typical synthesis, a solution of copper chloride (CuCl, 0.800 mmol), tris(diethylamino)phosphine (P(NEt_2_)_3_, 1.12 mmol), oleylamine (OAm, 3.93 mmol), and trioctylamine (TOA, 6.71 mL) was prepared such that OAm/P/Cu = 4.9/1.4/1.0 and [Cu] = 0.100 M. The solution was degassed under vacuum at 125 °C for 3 h and subsequently heated to a final temperature of *T*_f_ = 273 °C. This synthetic procedure was repeated a total of 8 times using nearly identical amounts and heating profiles (Table S1,Figure S1), yielding ensembles with equivalent sizes and LSPR energies. Unless otherwise noted, all samples were kept entirely anaerobic during synthesis, purification, and redox treatments.

**Figure 1:**
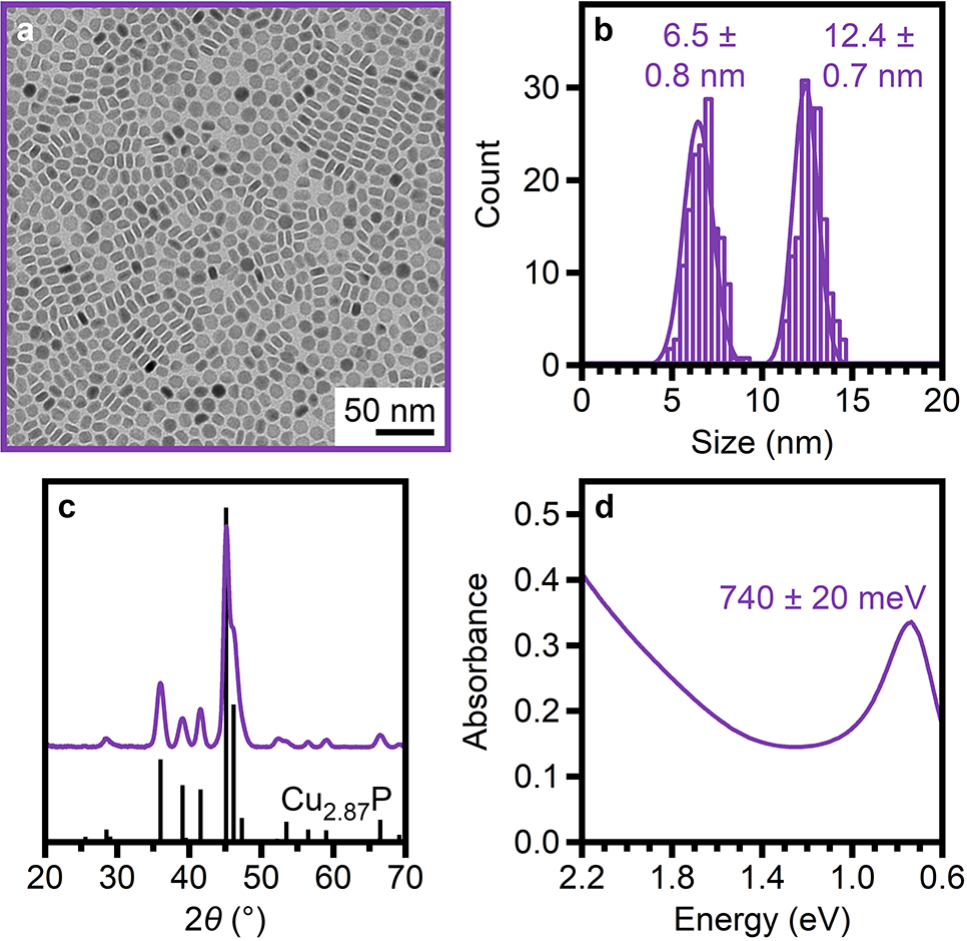
(a) TEM image, (b) statistical analysis of nanocrystal dimensions, (c) powder X-ray diffraction pattern (compared to that simulated for *P*6_3_*cm* Cu_2.87_P^12^), and (d) average absorption spectrum of Cu_3–*x*_P nanocrystals synthesized with [Cu] = 0.100 M, OAm/P/Cu = 4.9/1.4/1.0, and *T*_f_ = 273 °C (Synthesis 1).

Figure 1 presents the characterization of a typical ensemble of Cu_3–*x*_P nanocrystals synthesized herein (Synthesis 1). Transmission electron microscopy (TEM) imaging (Figure 1a) reveals an ensemble of nanoplatelets with a lateral dimension of 12.4 ± 0.7 nm and a height of 6.5 ± 0.8 nm (Figure 1b). This morphology is consistent with products of other colloidal nanocrystal syntheses^15−17,55−57^ and follows the crystal habit of the *P*6_3_*cm* phase of Cu_3–*x*_P.^12^ Powder X-ray diffraction (Figure 1c) confirms this phase and indicates a lateral dimension of 11.9 ± 0.1 nm and a height of 6.1 ± 0.1 nm by Scherrer analysis of the (300) (2θ = 45.092°) and (112) (2θ = 36.006°) reflections, respectively. The close agreement between sizes derived from TEM and powder X-ray diffraction suggests that the nanoplatelets are single-crystalline. Finally, the absorption spectrum (Figure 1d) contains a LSPR feature at 740 ± 20 meV, characteristic of Cu_3–*x*_P.^8,15−17^

Independent electronic absorption measurements on equal aliquots of the same Cu_3–*x*_P nanocrystal ensemble indicated non-negligible variation in the absorbance intensity (Figure S2). Although the concentrated Cu_3–*x*_P nanocrystal suspensions appeared as colloidal inks with no visible scattering or settling of solids, we hypothesize that aggregative platelet-stacking interactions led to suspension heterogeneity at the nanoscale, especially in more concentrated suspensions. Absorption spectra are thus shown as an average of three spectra to obtain more accurate intensities for quantitative optical analyses. To determine the yield, Cu_3–*x*_P nanocrystals were quantitatively digested in nitric acid and the Cu^2+^ content was determined using the ^2^E_g_ → ^2^T_2g_ absorption (Figure S3). The synthesis presented in Figure 1 had a yield of 75 ± 3% based on Cu, which is in agreement with yields determined by elemental analysis of similarly synthesized Cu_3–*x*_P nanocrystals.^16^ This yield was used to determine an extinction coefficient of ε_3.1 eV_^Cu^ = 2100 ± 200 L/((mol Cu) cm) for as-synthesized Cu_3–*x*_P nanocrystals. The yields of the remaining syntheses (Table S1) were determined using this extinction coefficient. The nearly identical heating profiles (Figure S1) enable the synthesis of Cu_3–*x*_P nanocrystal ensembles with reproducible LSPR absorption, which allows us to make comparisons between ensembles when evaluating the postsynthetic redox treatments (Scheme S1).

**Figure 2:**
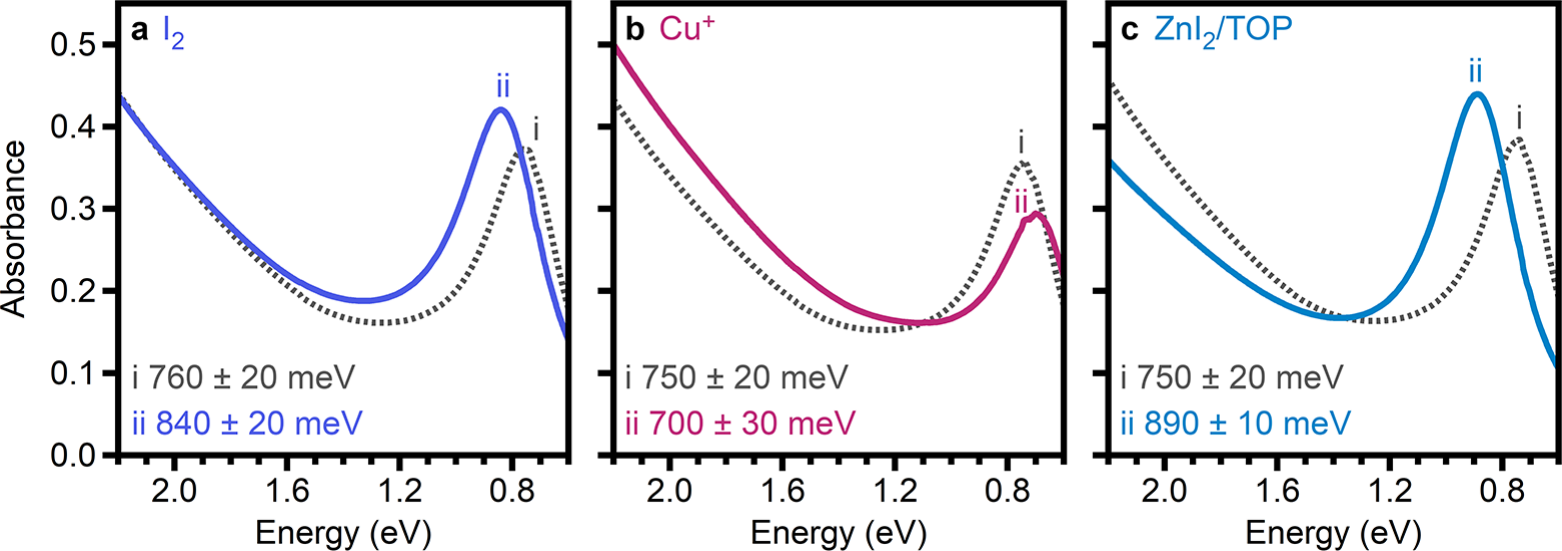
Average absorption spectra of Cu_3–*x*_P nanocrystals (i) before and (ii) after (a) oxidation with I_2_ (I_2_/Cu = 30), (b) reduction with Cu (Cu_Reductant_^+^/Cu_Nanocrystals_ = 0.24), and (c) oxidation with ZnI_2_/TOP (ZnI_2_/TOP/Cu = 3.8/15/1).

The first redox chemistry investigated was oxidation by I_2_, as it has been established to act as a Cu-coupled oxidant of copper chalcogenide nanocrystals.^40,53,54^ In contrast to previous studies in which I_2_ and nanocrystals were mixed as solutions on the cuvette scale, a toluene suspension of an entire batch of Cu_3–*x*_P nanocrystals (Synthesis 2) was oxidized with I_2_ under static vacuum for 20 h without direct contact (Figure S4). The oxidized Cu_3–*x*_P nanocrystals were purified by addition of acetonitrile (MeCN) and collected by centrifugation. After oxidation, the LSPR energy was blue-shifted by 80 meV compared to that of the as-synthesized ensemble (Figure 2a, Table S2), indicating an increase in the delocalized hole concentration. TEM images of the purified nanocrystals revealed that the size and morphology were unchanged after oxidation (Figure S5a). Powder X-ray diffraction indicated retention of the *P*6_3_*cm* Cu_3–*x*_P phase after oxidation with I_2_ (Figure S6a).

The purified nanocrystals were quantitatively digested in nitric acid, and absorption spectra of the resulting solutions (Figure S7a) were used to determine that the Cu content of the oxidized nanocrystals was 93 ± 10% of the sample prior to oxidation (Table S2), corresponding to a removal of 7% of Cu atoms from the nanocrystals. To corroborate this analysis, the supernatant collected during nanocrystal purification was evaporated and the resulting residue analyzed. Digestion and optical analysis of the residue indicated that 6.6% of Cu was removed from the Cu_3–*x*_P nanocrystals and reacted to form CuI (Section S1, Figure S8), consistent with the amount of Cu lost based on analysis of the digested nanocrystals. The oxidation process was repeated on another Cu_3–*x*_P ensemble (Synthesis NS2), and powder X-ray diffraction on the resulting supernatant residue revealed the presence of copper(I) iodide (CuI, Figure S9). Analogous CuI formation was reported during the Cu-coupled oxidation of copper sulfide nanocrystals.^40^ These results indicate that oxidation with I_2_ creates additional Cu vacancies that lead to delocalized holes without damaging the Cu_3–*x*_P nanocrystals.

The second redox treatment investigated was reduction with Cu^+^, as tetrakis(acetonitrile)copper(I) hexafluorophosphate ([Cu(MeCN)_4_]PF_6_) has been shown to act as a Cu-coupled reductant for copper chalcogenide nanocrystals.^28,31,52^ In a modification of a previously reported procedure,^31^ methanol (MeOH), OAm, and [Cu(MeCN)_4_]PF_6_ solid were added directly to a diluted Cu_3–*x*_P nanocrystal batch in toluene (Synthesis 3, [Cu_Cu3–*x*P_] = 0.035 M, MeOH/OAm/[Cu(MeCN)_4_]PF_6_/Cu_Cu3–*x*P_ = 7.84/0.48/0.24/1.00) and the resulting mixture was stirred at room temperature for 20 h. The reduced Cu_3–*x*_P nanocrystals were purified by precipitation with MeCN and collection via centrifugation. After reaction with Cu^+^, the LSPR energy was red-shifted by 50 meV compared to that of the as-synthesized ensemble (Figure 2b, Table S2). TEM revealed that the Cu_3–*x*_P nanoplatelet size and morphology were unchanged after reduction (Figure S5b), and powder X-ray diffraction showed that the *P*6_3_*cm* phase of Cu_3–*x*_P was maintained after reduction (Figure S6b). The purified nanocrystals were digested, and absorption spectroscopy (Figure S7b) was used to determine a postreduction Cu content of 94 ± 13% relative to the original ensemble (Table S2).

Reduction of copper sulfide nanocrystals with Cu^+^ has been shown to lead to equimolar production of Cu^2+^ in the reaction solution.^31^ To investigate this in our treatment, the supernatant was collected following purification and concentrated to give an oil. Surprisingly, electronic absorption spectroscopy of the oil (Figure S10ai) revealed no Cu^2+^. When this solution was exposed to air, Cu^2+^ was observed (Figure S10aii), indicating that the anaerobic supernatant contained primarily Cu^+^, which was oxidized in air. The supernatant oil was also analyzed by ^31^P and ^19^F NMR spectroscopy to probe the PF_6_^–^ environment (Figure S10b,c, respectively). When the oil was intentionally exposed to air, signal broadening was observed for the resonance in both the ^31^P and ^19^F spectra, corroborating the oxidation of diamagnetic Cu^+^ to paramagnetic Cu^2+^ upon air exposure. Quantitative analysis of the Cu^2+^ absorption feature (Section S2, Figure S11) indicated a loss of Cu relative to the amount of [Cu(MeCN)_4_]PF_6_ added to the solution. This loss suggests that Cu was inserted into the nanocrystals upon reduction, corresponding to an increase in Cu-content of 2.5%.

The third redox chemistry presented is an apparent oxidation that occurs when Cu_3–*x*_P nanocrystals are heated in the presence of both zinc iodide (ZnI_2_) and TOP. TOP has previously been shown to extract Cu from Cu_3–*x*_P nanocrystals.^8,9^ Additionally, Z-type ligands have recently been demonstrated to passivate hole traps of InP nanocrystal surfaces.^58^ In this treatment, a batch of Cu_3–*x*_P nanocrystals (Synthesis 4) was added to OAm, ZnI_2_, and TOP ([Cu] = 0.078 M, TOP/Zn/OAm/Cu = 16.0/4.0/3.0/1.0) and stirred at 100 °C for 20 h. The Cu_3–*x*_P nanocrystals were purified by precipitation with MeCN and collection via centrifugation. After reaction with ZnI_2_/TOP, the LSPR energy exhibited a blue shift of 140 meV relative to that of the as-synthesized ensemble (Figure 2c, Table S2). This blue shift is indicative of the generation of additional delocalized holes, consistent with oxidation of the nanocrystals. TEM of the treated Cu_3–*x*_P nanocrystals indicated that the size and morphology were unchanged (Figure S5c), and powder X-ray diffraction revealed that the Cu_3–*x*_P nanocrystals retained the *P*6_3_*cm* phase after oxidation (Figure S6c).

The purified nanocrystals were digested and analyzed, revealing a postredox Cu content of 74 ± 12% relative to the original ensemble (Figure S7c, Table S2). Again, the loss of Cu is consistent with an increase in delocalized hole concentration, as evidenced by absorption spectroscopy (Figure 2c). We note that some loss of Cu^+^ may be compensated by surface or intercalated Zn^2+^ (*vide infra*), which would not lead to extra delocalized holes. The supernatant collected during purification was concentrated, and the resulting oil was analyzed for Cu content. In this case, no Cu^2+^ could be detected by absorption spectroscopy, even after intentional air exposure (Figure S12i). This observation alone does not rule out the possibility of Cu-coupled oxidation and Cu-atom extraction, as it is likely that any Cu is ligated by TOP, keeping it as Cu^+^. This assertion is supported by the absence of Cu^2+^ absorption when a control solution containing CuI and TOP is exposed to air (Figure S12ii). Furthermore, ^31^P NMR spectroscopy of the supernatant suggest the presence of Cu (Section S3, Figure S13). The unchanged nanoplatelet size together with a statistically significant loss of Cu following treatment are indicators that oxidation using ZnI_2_/TOP is likely Cu-coupled. Additional evidence of Cu-atom extraction is provided by analysis of the structural data (*vide infra*).

Additional redox chemistries were explored but were considered only partially successful due to nanocrystal aggregation and/or unstable reduction. Specifically, stirring Cu_3–*x*_P nanocrystals with Li resulted in aggregation without any indication of reduction (Figure S14). The aggregation was eliminated when 1.5 OAm/Cu was included in the mixture, yielding nanocrystals in which the LSPR was red-shifted 90 meV relative to the original ensemble (Figure S15i). This red shift, however, spontaneously reversed upon removal of excess Li metal from the suspension (Figure S15ii), precluding reliable structural characterization of the reduced product. Additional oxidation chemistries, including treatment with cerium(IV) or ferrocenium, have been demonstrated for copper chalcogenide nanocrystals.^28,40^ Treatment of Cu_3–*x*_P nanocrystals with these oxidants leads to a blue shift in the LSPR energy (Figure S16i) but was accompanied by aggregation that was visible by eye as well as noticeable in the post-treatment TEM images (Figure S16ii). These treatments were thus not extended beyond the cuvette scale, and our studies focused only on the first three described treatments (oxidation with I_2_, reduction with Cu^+^, and oxidation with ZnI_2_/TOP).

**Figure 3:**
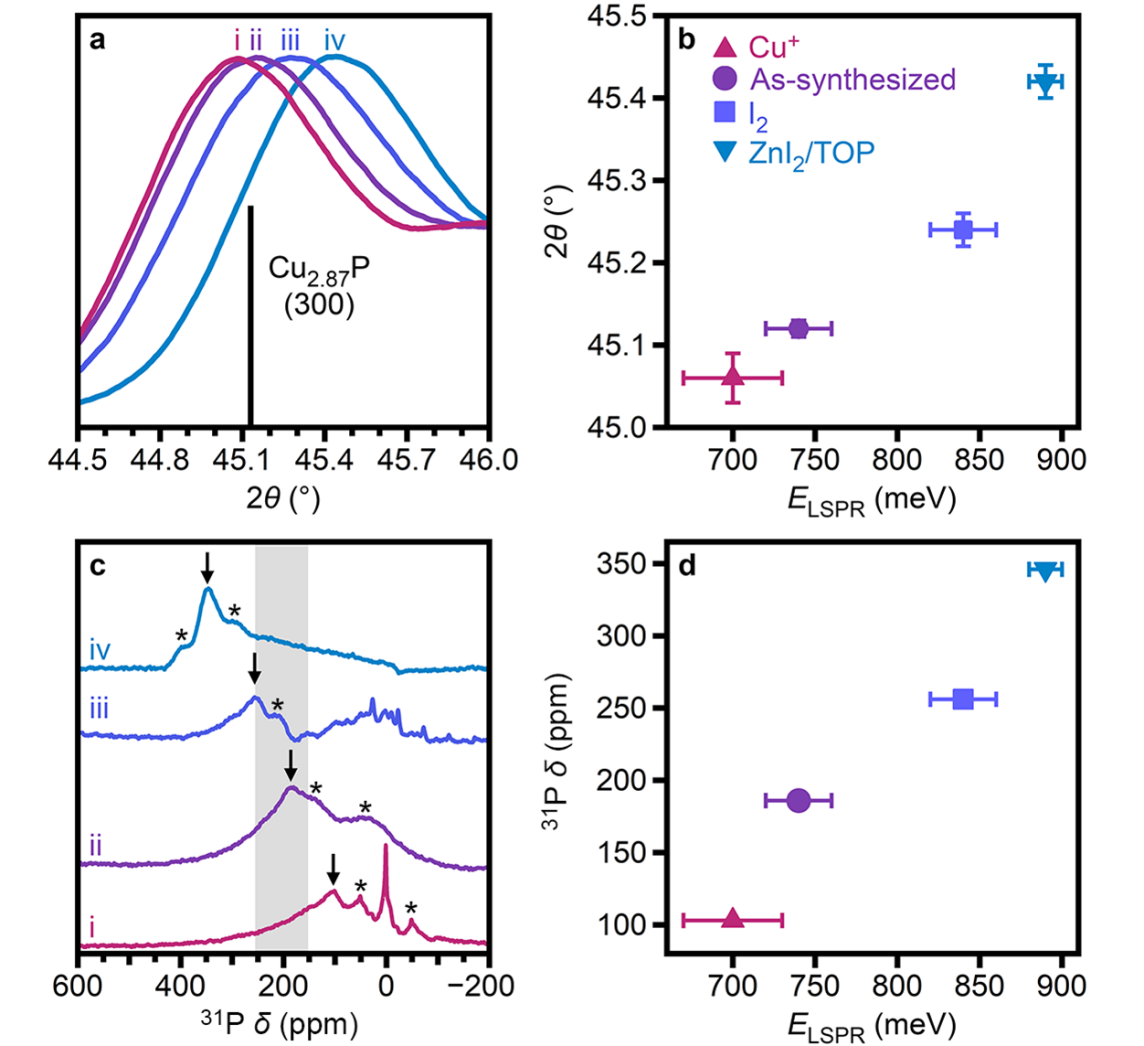
(a) Magnified powder X-ray diffraction patterns of Cu_3–*x*_P nanocrystal ensembles (i) reduced with Cu^+^, (ii) as-synthesized, (iii) oxidized with I_2_, and (iv) oxidized with ZnI_2_/TOP. The (300) reflection simulated from single-crystal *P*6_3_*cm* Cu_2.87_P^12^ is shown for comparison. (b) Corresponding 2θ values plotted as a function of the LSPR energy for the same samples. (c) Solid-state ^31^P MAS NMR spectra of the same ensembles. The shaded region corresponds to the range reported for bulk Cu_3–*x*_P.^1^ Asterisks indicate the spinning sidebands. The arrows indicate the chemical shifts of the core resonance. (d) Corresponding core chemical shifts plotted as a function of the LSPR energy for the same samples.

The increase/decrease in Cu content has been shown to correlate with a lattice expansion/contraction in substoichiometric copper chalcogenide nanocrystals^21,25,28,29^ and bulk Cu_3–*x*_P.^1,59^ As the aforementioned redox treatments are likely Cu-coupled, nanocrystals with higher LSPR absorption energies should have smaller unit-cell volumes, leading to a shift of the X-ray reflections to higher values of 2θ. Figure 3a shows the (300) reflection for the as-synthesized and redox-treated Cu_3–*x*_P nanocrystals. Importantly, for nanocrystals that are expected to contain less Cu (i.e., are more oxidized), this reflection shifts to higher 2θ (Figure 3b), indicating a lattice contraction. Calculations using the (003) and (2–12) reflections indicate that the lattice parameters, *a* and *c*, are smaller for nanocrystals that show a higher-energy LSPR and thus are expected to have a higher concentration of delocalized holes (Table 1, Figure S17). The range of observable values is larger for *a* than for *c* (Table 1, Figure S17), consistent with previously reported structural data for bulk Cu_3–*x*_P.^12,59^ Notably, the calculated unit-cell volumes attainable here range from 295.2 to 300.9 Å^3^ (Table 1), indicating that the nanocrystals can host compositions that are more copper-deficient or more copper-rich than have been reported in bulk *P*6_3_*cm* Cu_3–*x*_P (295.9–300.3 Å^3^).^1,12^

**Table 1:**
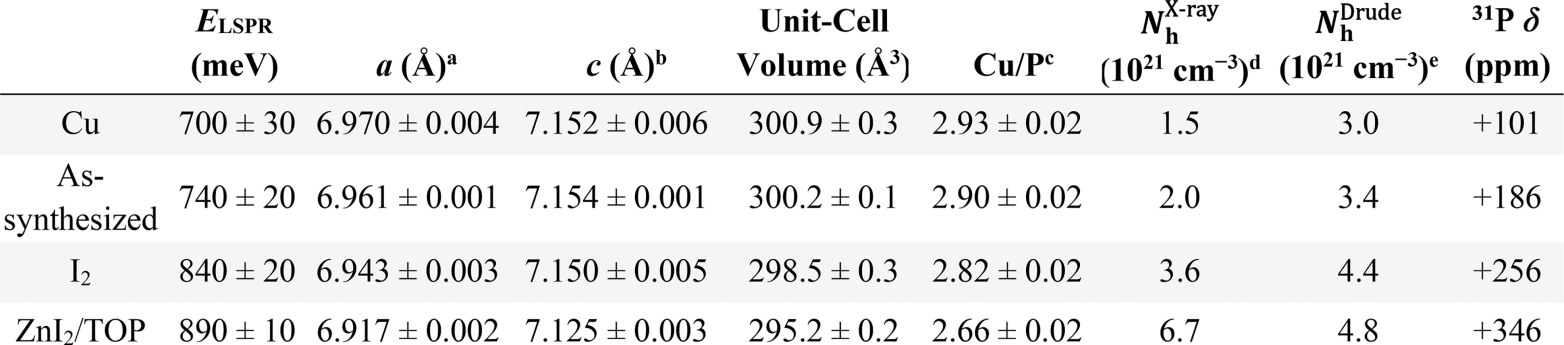
Characterization and Structural Analysis of As-Synthesized and Redox-Treated Cu_3–*x*_P Nanocrystals

a Calculated using the *d* spacing of the (300) reflection at 2θ ≈ 45°.

b Calculated using the *a* parameter and the *d* spacing of the (2–12) reflection at 2θ ≈ 36°.

c Estimated using the relationship between unit-cell volume and Cu/P for bulk Cu_3–*x*_P (Figure S18).

d Estimated from Cu/P, assuming one h^+^ per Cu vacancy.

e Estimated from optical data and modeling (Section S4).

As the lattice contraction is expected to depend directly on the number of Cu vacancies, the powder X-ray diffraction data can be used to extract compositional information, as has been demonstrated for Cu/Se ratios in substoichiometric copper selenide nanocrystals.^25^ Unlike methods that rely on elemental analysis of digested nanocrystal samples, compositional analysis based on diffraction data should reflect only the crystalline domain of the nanocrystal core and neglect contributions from amorphous surface regions. In other words, the diffraction data are expected to largely be blind to whether the nanocrystal surfaces are Cu- or P-rich and therefore provide vacancy concentrations that are representative of the nanocrystal core. To extract compositional data, the relationship between unit-cell volume and Cu/P was derived empirically from values previously reported for bulk Cu_3–*x*_P (Figure S18).^12^ This relationship was used to determine Cu/P values of the as-synthesized and redox-treated nanocrystals (Table 1). The estimated Cu/P range of 2.66–2.93 is consistent with that predicted by DFT calculations (2.67–2.83),^8^ particularly at the copper-deficient limit. If it is assumed that each vacancy yields one delocalized hole, the corresponding carrier density range is *N*_h_^X-ray^ = 1.5–6.7 × 10^21^ cm^–3^ (Table 1). For comparison, simplified Drude–Lorentz calculations (Section S4) were used to estimate carrier concentrations from the absorption spectra of the same samples (Figures 1d, 2). This analysis yields a narrower range of carrier densities (Table 1, *N*_h_^Drude^ = 3.0–4.8 × 10^21^ cm^–3^). Both ranges of values are in close agreement with carrier densities determined from Hall measurements on bulk Cu_3–*x*_P films (*N*_h_ = 3.8 × 10^21^ cm^–3^).^60^

Solid-state NMR spectroscopy has recently been demonstrated as a sensitive technique for probing delocalized carriers in degenerately doped semiconductor nanocrystals.^25,61^ For example, solid-state ^77^Se NMR of Cu_2–*x*_Se nanocrystals showed a downfield Knight shift with increased copper vacancies. The temperature dependence of the spin–lattice relaxation times followed a Korringa relationship,^62^ allowing estimation of the carrier densities, and therefore Cu vacancies, even when LSPR absorption was not observable.^25^ To our knowledge, nanoscale Cu_3–*x*_P has not yet been characterized by solid-state ^31^P NMR, but an analogous downfield Knight shift for increased copper vacancies^1,59^ as well as Korringa behavior^63^ have been demonstrated for bulk Cu_3–*x*_P. Here, we use solid-state ^31^P MAS NMR spectroscopy to provide an additional correlation of the structural and optical changes in as-synthesized and redox-treated Cu_3–*x*_P nanocrystals (Figure 3c,d). As expected, the nanocrystal spectra are significantly broadened relative to those of the bulk.^1,59^ Furthermore, additional, often sharper resonances are present in the spectra of redox-treated nanocrystals, likely due to contributions from residual molecular redox and surface P-species. In the spectrum of the as-synthesized Cu_3–*x*_P nanocrystals (Figure 3cii), the most intense resonance at δ = +186 ppm falls within the reported chemical shift range for bulk Cu_3–*x*_P.^1,59^ Additional intensity is due to spinning sidebands (indicated by asterisks), which preclude the identification of surface P resonances. Saturation–recovery experiments performed at 186 ppm and 300 K fit well to a single exponential (Figure S19), yielding a spin–lattice relaxation time of *T*_1_ = 15.8 ± 0.7 ms. This value agrees well with that reported for bulk Cu_3–*x*_P (*T*_1_ = 16 ms at room temperature).^63^ We thus assign the broad intense resonance indicated by the arrows (Figure 3c) to the nanocrystal “core”.

Similar to the empirically derived relationship between unit-cell volume and Cu/P mentioned above, we can derive a relationship between the ^31^P chemical shift and unit-cell volume based on values reported for bulk Cu_3–*x*_P (Figure S20).^1,59^ This empirical relationship can be used to determine the expected chemical shift of our samples based on the unit-cell volume determined by X-ray diffraction (Table S3). As the expected chemical shifts are close in value to the most intense resonance in each of the spectra (Table S3), the most intense resonance was assigned as the isotropic chemical shift for the nanocrystal core in each treatment. The shift of the core resonance of redox-treated nanocrystals is consistent with the Knight shift behavior observed in bulk Cu_3–*x*_P;^1,59^ specifically, nanocrystals that are more oxidized, and thus have higher carrier densities, display a more downfield shift of the core resonance (Figure 3c,d; Table 1). Saturation–recovery experiments, however, did not fit well to a single exponential (Figure S21), likely due to the presence of additional species formed during redox treatments. This non-single-exponential behavior precludes the extraction of reliable spin–lattice relaxation times that would provide independent estimates of the delocalized hole concentration.^25,64−66^

**Figure 4:**
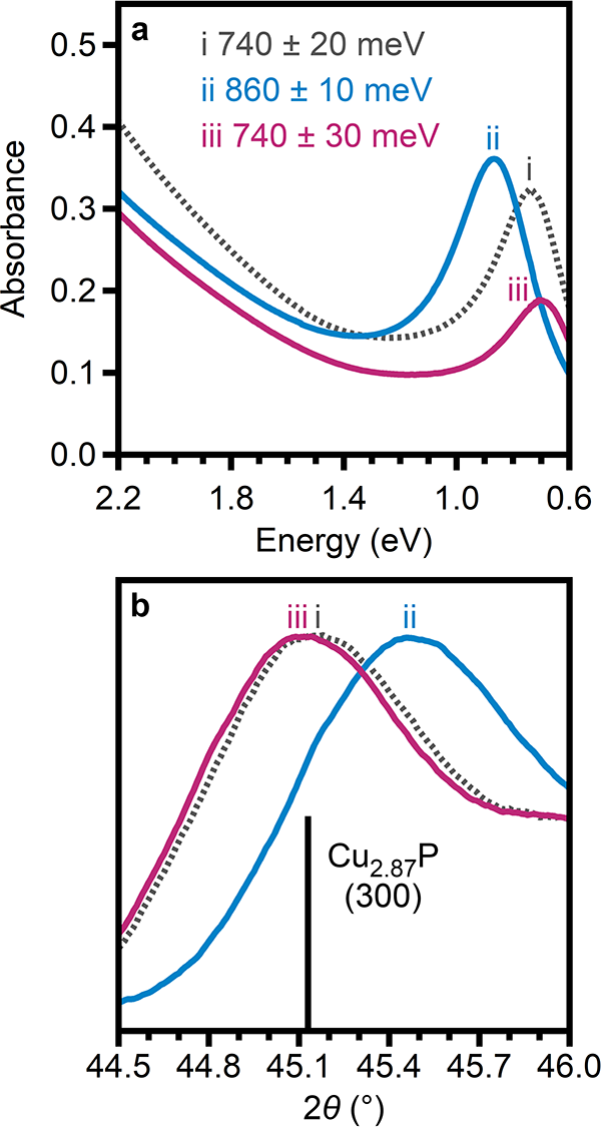
(a) Absorption spectra and (b) magnified powder X-ray diffraction patterns (compared to that simulated for *P*6_3_*cm* Cu_2.87_P^12^) for (i) as-synthesized Cu_3–*x*_P nanocrystals and (ii) the same nanocrystals oxidized with ZnI_2_/TOP and then (iii) re-reduced with Cu^+^.

An important feature of electronically doped nanocrystals is the potential for reversible tuning of the carrier density. To test the reversibility of Cu_3–*x*_P oxidation and reduction, we performed a series of experiments in which the same ensemble was oxidized, then reduced, or vice versa (Scheme S2). To demonstrate reversibility of the oxidative treatments, Cu_3–*x*_P nanocrystals were oxidized with I_2_ or ZnI_2_/TOP, purified via washing, and rereduced with Cu^+^. Figure 4 and Figure S22 present characterization that demonstrates the reversibility of oxidation with ZnI_2_/TOP. Initial oxidation led to a blue shift of the LSPR from 740 ± 20 to 860 ± 10 meV (Figure 4ai,ii). Subsequent reduction with Cu^+^ yielded a red shift of the LSPR to 700 ± 20 meV (Figure 4aiii). This final LSPR energy is consistent with that observed when as-synthesized nanocrystals were reduced with Cu^+^ (Figure 2b). Similarly, the oxidation and rereduction are accompanied by a lattice contraction and re-expansion (Figure 4b), finishing with a unit-cell volume equivalent to that of nanocrystals treated only with Cu^+^ (Table S4). TEM and powder X-ray diffraction show that the size, morphology, crystallinity, and *P*6_3_*cm* phase of the nanocrystals are retained throughout oxidation and rereduction (Figure S22, Table S5). These observations indicate that oxidation with ZnI_2_/TOP is fully reversible. An analogous experiment demonstrated reversibility when nanocrystals were oxidized with I_2_ and rereduced with Cu^+^. These treatments also led to a reversible LSPR shift (Figure S23a) as well as a lattice contraction and re-expansion (Figure S23b, Table S5). Powder X-ray diffraction (Figure S23c) and TEM imaging (Figure S23d,e) again showed that nanocrystal crystallinity, size, and morphology were unchanged during oxidation and rereduction.

Surprisingly, when the order of redox treatments was switched (reduction followed by reoxidation), the reversibility was not maintained. When Cu_3–*x*_P nanocrystals were reduced with Cu^+^, then oxidized with I_2_, the absorption significantly decreased (Figure S24a) and powder X-ray diffraction revealed decreased crystallinity (Figure S24b), suggesting degradation of the Cu_3–*x*_P nanocrystals. TEM images showed decreased contrast for the Cu_3–*x*_P nanocrystals (Figure S24c), although no significant decrease in size was observed (Figure S24d). When Cu_3–*x*_P nanocrystals were reduced with Li in the presence of excess OAm and reoxidized with I_2_, even greater degradation was observed, as evidenced by nearly complete loss of absorption in the near-IR (Figure S25a) and of X-ray diffraction (Figure S25b). These findings indicate that, while changes to the “core” of the Cu_3–*x*_P nanocrystals are in principle reversible, incompatibility between successive redox experiments can lead to nanocrystal degradation, likely due to altered surfaces and leftover impurities.

To understand the range of carrier densities accessible with these redox treatments, we evaluated whether the extent of oxidation or reduction was sensitive to the starting composition/properties of the as-synthesized Cu_3–*x*_P nanocrystals. We have previously demonstrated that Cu_3–*x*_P nanocrystals with a higher-energy LSPR can be synthesized using a lower temperature to decrease the reactivity during nucleation.^16^ Following this method, a lower reaction temperature (20 h at *T* = 200 °C, Figure S26a) with the standard composition ([Cu] = 0.100 M, OAm/P/Cu = 4.9/1.4/1.0, Synthesis NS1) was used to synthesize Cu_3–*x*_P nanocrystals with the LSPR absorbance at 880 ± 20 meV (Figure S26bi). This LSPR energy and the (300) reflection at a higher 2θ (45.25°, Figure S26ci) suggest that these nanocrystals are more Cu-deficient than those synthesized with identical precursor composition but at higher temperature. Treatment of these “Cu-deficient” nanocrystals with ZnI_2_/TOP did not yield any further blue shift of the LSPR absorption, although some loss of intensity was observed (Figure S26bii). Examination of the (300) reflection revealed a shift to higher 2θ following treatment with ZnI_2_/TOP (Figure S26cii), despite the unchanged LSPR energy. This apparent lattice contraction and decreased absorption intensity suggest that some additional Cu vacancies were formed but were not compensated by the formation of delocalized holes. These “inactive” defects are likely also present in the standard Cu_3–*x*_P nanocrystals oxidized with ZnI_2_/TOP (Figure 3aiv), although to a lesser extent. In contrast, upon reduction with Cu^+^, the LSPR energy red-shifted substantially to 760 ± 20 meV (Figure S26biii). Powder X-ray diffraction (Figure S26c) and TEM imaging (Figure S27) showed that the crystallinity, size, and morphology of the nanocrystals remained unchanged following both treatments. These observations are consistent with the expectation that the level of achievable oxidation/reduction is limited by the redox potential of the treatment rather than by the initial nanocrystal Fermi level^18,67^ (i.e., for a given redox treatment, the level of achievable oxidation/reduction is limited by *E*_LSPR_^final^ and *N*_h_^final^, rather than by Δ*E*_LSPR_ and Δ*N*_h_).

**Figure 5:**
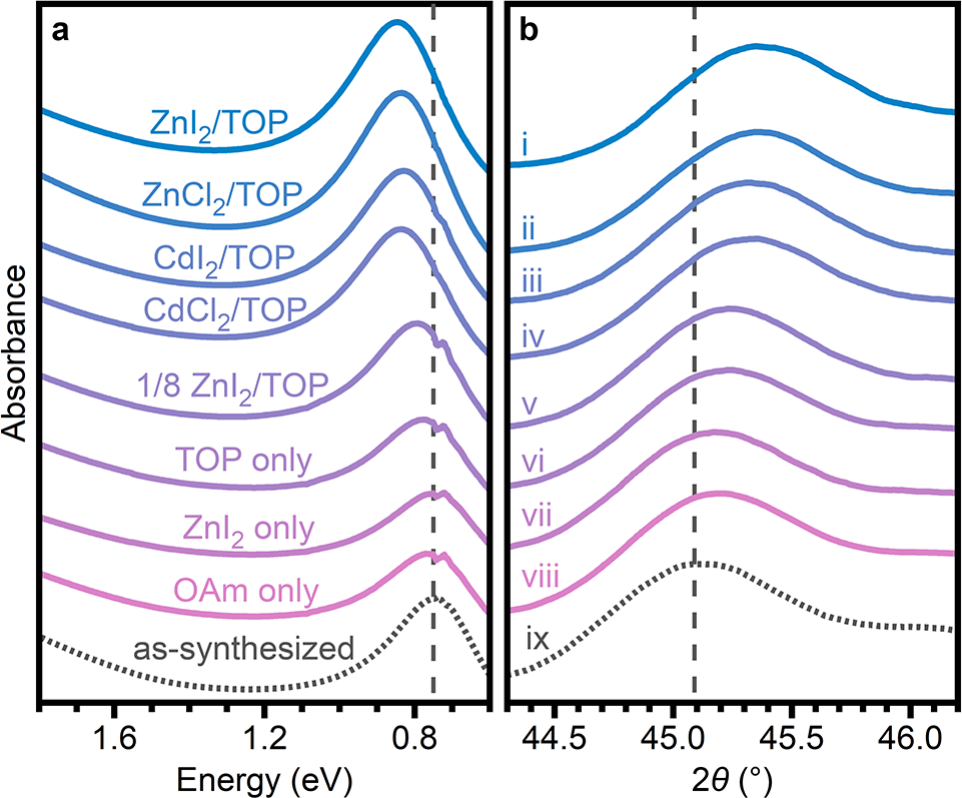
(a) Absorption spectra and (b) magnified powder X-ray diffraction patterns of Cu_3–*x*_P nanocrystals treated with (i) ZnI_2_/TOP, (ii) ZnCl_2_/TOP, (iii) CdI_2_/TOP, (iv) CdCl_2_/TOP, (v) ZnI_2_/TOP (1/8), (vi) TOP only (no MX_2_), (vii) ZnI_2_ only (no TOP), and (viii) OAm only. Dashed lines correspond to the values for the (ix) as-synthesized nanocrystals. Details of the experimental conditions are provided in Table S6.

Although oxidation with ZnI_2_/TOP provides the greatest level of oxidation, the mechanism for doing so is unclear. The correlation between decreasing unit-cell volume (and estimated Cu/P), increasing LSPR energy and increasingly downfield ^31^P core resonance (Figure 3, Table S4) suggest that the ZnI_2_/TOP treatment results in a Cu-coupled oxidation. This hypothesis is further supported by the reversibility of the ZnI_2_/TOP treatment by reduction with Cu^+^ (Figure 4). It is likely that Cu-atom removal is facilitated by TOP coordination to form copper(I) phosphine molecular complexes, as TOP is known to extract Cu from Cu_3–*x*_P nanocrystals.^8,9^ It is not expected, however, that either ZnI_2_ or TOP is capable of oxidizing Cu_3–*x*_P nanocrystals. To learn more about this treatment, a series of experiments were performed to assess the importance of the identity of MX_2_ (altering M and X; M^2+^ = Zn^2+^, Cd^2+^; X^–^ = Cl^–^, I^–^, OAc^–^) and of the amount of MX_2_/TOP added (decreasing the equivalents of ZnI_2_ and TOP, including omission of each). Detailed conditions of the various treatments are provided in Table S6. All treatments used the same ensemble (Synthesis 7) and were heated in parallel at 100 °C for 20 h. TEM (Figure S28) and powder X-ray diffraction (Figure S29) showed that nanocrystal size, morphology, and phase were maintained following the MX_2_/TOP treatments. Figure 5 presents the absorption spectra and magnified powder X-ray diffraction patterns for the as-synthesized ensemble as well as the nanocrystals following various treatments. The largest increase in both LSPR energies and 2θ values (indicating most Cu-deficient) were observed with treatments that contained both MX_2_ and TOP (MX_2_/TOP/Cu ≈ 4/16/1) and were independent of MX_2_ identity (Figure 5i–iv, Table S6). The observation of comparable shifts with either ZnX_2_ or CdX_2_ suggests that M^2+^ intercalation is not contributing significantly to the changes, as the larger Cd^2+^ is not expected to intercalate. These increases were smaller when less ZnI_2_/TOP was used (ZnI_2_/TOP/Cu ≈ 0.5/2/1, Figure 5v, Table S6). When MX_2_ or TOP was omitted entirely, the absorption spectra did not shift from the as-synthesized sample, but the (300) reflection was shifted to higher 2θ (Figure 5vi,vii, respectively, and Table S6). These slight lattice contractions may indicate that (1) TOP alone can extract a small amount of Cu from the Cu_3–*x*_P nanocrystals and (2) a small amount of Cu can be replaced by Zn, even when complete cation exchange does not proceed. We note, however, that a very minor contraction is observed without the presence of MX_2_ or TOP (OAm only, Figure 5viii, Table S6), indicating that a small amount of Cu may be annealed out under these conditions. Treatment with Zn(OAc)_2_/TOP led to irreversible aggregation of the nanocrystals, precluding analysis of the LSPR absorption. Powder X-ray diffraction, however, revealed a lattice contraction comparable to the treatment using small amounts of ZnI_2_/TOP (Table S6). We also note that for nanocrystals oxidized with the zinc halides and TOP, an additional unassigned reflection was observed in the X-ray diffraction pattern (Figures S6c, S22cii, S29).

To gain insight into the role of the divalent metal salt, select MX_2_/TOP-treated nanocrystals were further characterized using mass-spectrometry-detected inductively coupled plasma (ICP–MS) and scanning TEM with energy dispersive X-ray spectroscopy (STEM–EDS) mapping. These measurements were performed on nanocrystals treated with MI_2_/TOP (Figure 5i,iii) to avoid the confounding effect of Cl^–^, which is likely present on the nancrystal surface from synthesis.^58,68^ ICP–MS on dried, purified nanocrystal solids revealed that oxidations with both CdI_2_/TOP and ZnI_2_/TOP led to a loss of Cu from the nanocrystals of ∼15% (Table S7). Additionally, the amounts of Zn or Cd retained after washing are comparable to the amounts of extracted Cu (M/ΔCu = 0.5–0.9, Table S7). STEM–EDS mapping was used to determine the spatial distribution of Zn, Cd, and I atoms in Cu_3–*x*_P nanocrystal ensembles oxidized with MI_2_/TOP. As expected for the as-synthesized sample, there was colocalization of the Cu Kα signal with the nanoplatelets and an absence of Zn Kα, Cd Lα, and I Lα signals above the noise limit (Figure S30). STEM–EDS mapping of the nanocrystals oxidized with ZnI_2_/TOP revealed the presence of nanoscale domains of Zn and I orthogonally localized with respect to the Cu Kα signal and the Cu_3–*x*_P nanocrystals (Figures S31, S32). These regions may correspond to the additional species detected in the powder X-ray diffraction patterns of nanocrystals treated with zinc halides (Figure S29). In contrast, STEM–EDS mapping of the nanocrystals oxidized with CdI_2_/TOP revealed colocalization of both Cd Lα and I Lα signals with the Cu_3–*x*_P nanocrystals and no indication of separate domains (Figures S33, S34). Unfortunately, higher-resolution, single-particle mapping was not possible due to degradation of the nanocrystals by the electron beam.

## Discussion

The data presented in this study show that solution-phase redox chemistries can be leveraged to postsynthetically tailor the plasmonics of colloidal Cu_3–*x*_P nanocrystals on the scale of an entire synthesis ensemble. This postsynthetic tuning represents a valuable extension of the LSPR control that was previously demonstrated through modulation of the reactivity in aminophosphine-based syntheses.^16^ Anaerobic chemistries for tuning the LSPR are especially important for Cu_3–*x*_P nanocrystals, as metal phosphide surfaces have been shown to readily form a substantial amount of oxidized phosphate defects.^69^ Indeed, when Cu_3–*x*_P nanocrystals were stored aerobically, heavy oxidation occurred, as indicated by the observation of lower-contrast shells in TEM images (Figure S35). The three highlighted anaerobic redox chemistries leave the Cu_3–*x*_P nanocrystal crystallinity, size, and morphology unchanged. Additionally, crystallite sizes estimated by Scherrer analysis of powder X-ray diffraction data (Table S8) indicate that nanoparticle single crystallinity remains intact after redox treatments.

Two of the Cu-coupled redox chemistries investigated in this study, reduction with Cu^+^ and oxidation with I_2_, have previously been demonstrated with an analogous class of nanocrystals, the copper chalcogenides.^28,31,40,52−54^ The method for oxidation with I_2_, however, was adapted to a larger scale (>10 times those previously reported). Furthermore, the previously unreported oxidation with ZnI_2_/TOP led to the greatest change in Cu-vacancy concentration, LSPR absorption energy, and ^31^P chemical shift (Figure 3, Table 1), indicating the largest modulation of the carrier density.

### Correlations among Optical, Electronic, and Structural Properties

**Figure 6:**
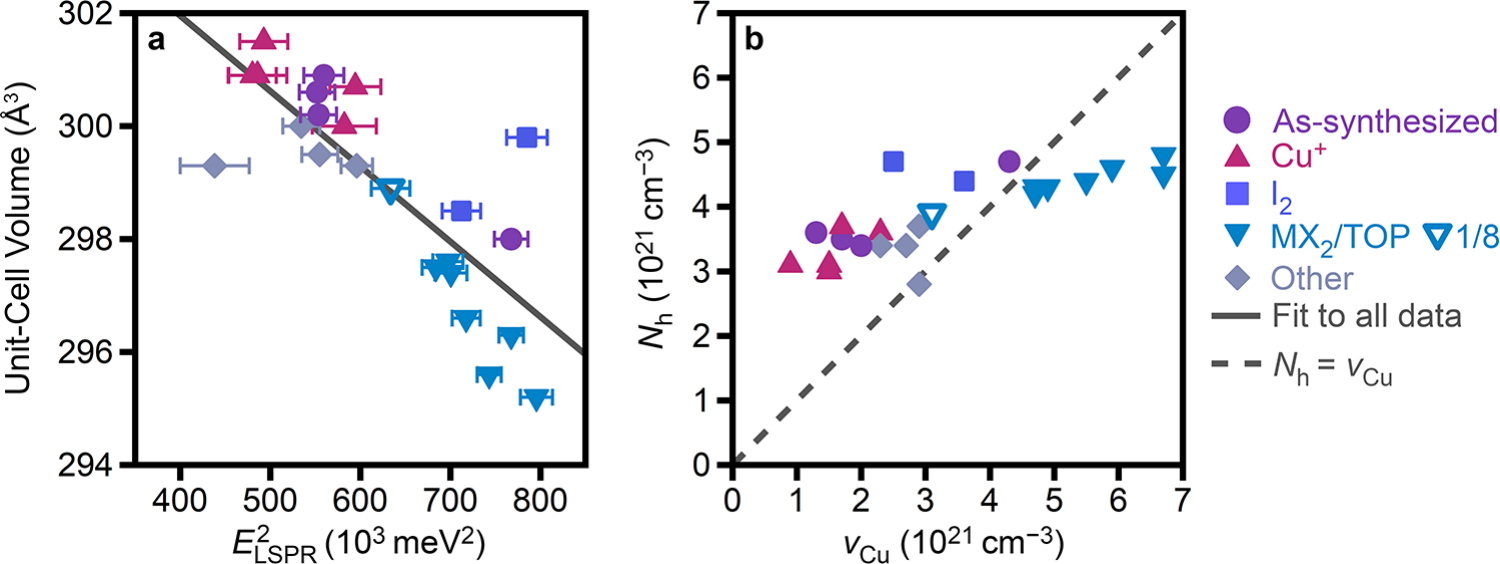
(a) Unit-cell volume as a function of square of LSPR maximum. The solid gray line is a fit to all collected data. (b) Delocalized hole concentration (*N*_h_) estimated from a simplified Drude model plotted as a function of the Cu vacancy concentration (*v*_Cu_) estimated from X-ray diffraction. The dashed gray line shows *N*_h_ = *v*_Cu_._._

LSPR absorption is an emergent phenomenon with nanoscaling, and thus there is no previously reported quantitative correlation between the LSPR absorption and the composition of Cu_3–*x*_P. Building a framework for the optical–structural correlation is valuable for understanding the factors enabling tunability of the LSPR absorption, including elucidating the influence of potential surface or other localized states that may lead to inactive vacancies. From the many samples characterized herein, there is an approximately linear relationship between the unit-cell volume and *E*_LSPR_^2^ (Figure 6a). This trend is expected because an increase in Cu^+^ vacancies, and concomitant contraction of the unit cell, should lead to the introduction of more delocalized holes for charge-compensation. According to the Drude–Lorentz model,^44,70−72^ this increase in *N*_h_ increases the LSPR energy as *E*_LSPR_^2^ ∝ *N*_h_ (eqs S5, S6). Notably, the samples treated with MX_2_/TOP are systematically to the left of the linear fit for unit-cell volume vs *E*_LSPR_^2^ (Figure 6a), meaning that the LSPR energy is lower than expected for a given unit-cell volume. Related to this observation, when “Cu-deficient” nanocrystals were treated with MX_2_/TOP, X-ray diffraction revealed a lattice contraction without a concomitant blue shift in the LSPR energy. Similarly, Cu_3–*x*_P nanocrystals treated with either TOP or ZnI_2_ (but not both) exhibited a lattice contraction without changes to the LSPR energy (Figure 5vi,vii). Together, these results may indicate that treatment with MX_2_/TOP leads to a greater portion of Cu vacancies being compensated by surface species (possibly containing M^2+^) rather than by delocalized holes. Although intercalation by Zn^2+^ may be possible, the observation of similar changes when using Cd^2+^ salts suggests that intercalation is not significant. The deviation from the linear fit is greater at smaller unit-cell volumes (more vacancies), suggesting that this effect may be due to the large number of vacancies, and not necessarily an effect of the oxidation with MX_2_/TOP in particular. Data labeled “other” are from the unsuccessful or partially successful redox treatments.

Figure 6b plots the delocalized hole concentration (*N*_h_) estimated from a simplified Drude model (Section S4) as a function of the Cu vacancy concentration (*v*_Cu_) estimated from powder X-ray diffraction. The dashed line is *N*_h_ = *v*_Cu_. At lower *v*_Cu_, the Drude model overestimates *N*_h_ relative to *v*_Cu._ Overestimation of the carrier concentration from a simplified Drude model has been noted in various plasmonic semiconductor nanocrystals, including anisotropic Cu_2–*x*_Te nanocrystals,^73^ and can arise from an inhomogeneous distribution of defects^74,75^ or nonparabolic dispersion of the valence band.^61,76^ At higher *v*_Cu_, deviation from the Drude model suggests that not all vacancies result in delocalized holes. As discussed previously, this is likely due to compensation by localized/surface species at higher *v*_Cu_. In particular, for nanocrystals treated with MX_2_/TOP, only those at higher *v*_Cu_ fall below *N*_h_ = *v*_Cu_, again suggesting that this effect may be due to the large number of vacancies, rather than the MX_2_/TOP chemistry.

The trend relating unit-cell volume and ^31^P chemical shift (Figure S20) agrees well with values reported for bulk counterparts,^1,59^ indicating that nanoscaling, to the extent demonstrated in this study, does not profoundly alter the electronic or structural properties of Cu_3–*x*_P.

### Oxidation with MX_2_ and TOP

As treatment with MX_2_/TOP produces the most profound change in Cu_3–*x*_P structural and optical properties, an understanding of the redox mechanism could enable extension to greater carrier densities or to other plasmonic nanomaterials. It is likely that the oxidation is Cu-coupled based on the decrease in Cu content with blue-shifting LSPR and decreasing unit-cell volume (Figure S36, Table S9). A possible mode of Cu extraction is Cu leaving the nanocrystal as a TOP-ligated copper(I) complex with a halide counteranion, which may come from either MX_2_ or the Cl^–^ already on the nanocrystal surface from synthesis.^58,68^ This idea is supported by a previous observation that Cu-atom extraction from Cu_3–*x*_P nanocrystals during cation exchange was controlled by the relative amount of TOP.^9^ The component of the oxidation mechanism with no simple explanation is the loss of electrons from the nanocrystals. It seems that MX_2_ is required for oxidation, as reaction with just TOP and/or OAm does not induce the full LSPR blue shift (Figure 5avi,viii). Furthermore, elemental analysis reveals the presence of Zn and Cd atoms in the purified postoxidation nanocrystal samples in amounts consistent with the amount of extracted Cu (which only corresponds to 2–3% of total MX_2_ used in the reaction, Table S7). It is unclear, however, if MX_2_ is directly involved in oxidation or only provides charge compensation. Unlike the oxidation with I_2_, the halogen atoms in the oxidation treatment with MX_2_/TOP are anionic prior to redox, and thus halogen reduction can be ruled out during treatment with MX_2_/TOP.

One possible explanation for the increase in delocalized hole concentration with the MX_2_/TOP treatment is that MX_2_ binds undercoordinated P atoms at the nanocrystal surface as a Z-type ligand. There is precedent for this in a recent study, which posits the existence of Z-type ligation of aminophosphine-synthesized indium phosphide nanocrystals after reaction with ZnCl_2_ at 100 °C.^58^ The same study used DFT calculations to reveal that P-atom passivation by ZnCl_2_ eliminates hole surface traps. If this phenomenon translates to Cu_3–*x*_P nanocrystals, it could explain why reaction with MX_2_ leads to an increase in delocalized hole carrier concentration, especially in the presence of Cu-extracting TOP that exposes additional undercoordinated surface P atoms. Experiments to probe surface association of MX_2_ by STEM–EDS elemental mapping (Figures S31–S34) were inconclusive. Although Cu_3–*x*_P nanocrystals oxidized with CdI_2_/TOP have Cd colocalized with the nanocrystals (Figures S33, 34), ligand-shell physisorption of Cd-containing species could not be ruled out due to the inability to measure at higher-resolution without destroying the nanocrystals. An additional confounding result is the observation of Zn-containing (and Cu-free) nanoscale domains in the STEM–EDS mapping of ZnI_2_/TOP-oxidized nanocrystals (Figures S31, S32). It is unlikely that the domains are unreacted zinc halide, as the additional diffraction peak observed with ZnX_2_-treated samples (X^–^ = Cl^–^, I^–^; Figures S6c, S22cii, S29) does not match any known phase of ZnCl_2_ or ZnI_2_. Although the additional peak nearly coincides with the (220) reflection of α-Zn_3_P_2_,^77^ other reflections of α-Zn_3_P_2_ were not observed. This impurity reflection also does not match known phases of Cu(0), Zn(0), or copper halide salts. The fact that the diffraction peak is observed also in the absence of TOP and with minimal nanocrystal oxidation (Figure S29) suggests that the corresponding species is probably generated by a nonproductive side reaction.

### Practical Considerations for Solution-Phase Redox Chemistries

Some key limitations of the solution-phase Cu_3–*x*_P nanocrystal redox chemistries presented in this study include coprecipitation of insoluble species during purification as well as nanoplatelet aggregation. Insoluble molecular impurities coprecipitated with Cu_3–*x*_P nanocrystals during purification after reduction with Cu^+^. This was determined by the observation of a substantially larger powder mass during sample preparation for solid-state ^31^P MAS NMR, as well as the sharp resonances in the corresponding measurement near 0 ppm (Figure 3ci). These molecular impurities may impede reversibility of reduction with Cu^+^ (Figure S24).

Irreversible aggregation of Cu_3–*x*_P nanocrystals was most problematic with the Li treatment (Figure S14). The presence of LiCl impurities in the powder X-ray diffraction following treatment with Li (Figure S14b) suggests that adventitious species in the reaction mixture oxidize Li, which may remove Cl^–^ from the Cu_3–*x*_P nanocrystal surface to form LiCl. The existence of Cl^–^ as a surface-bound species is inferred from its precedent as a copassivant, along with OAm, on the surface of metal chloride and aminophosphine synthesized metal phosphides.^58,68^ The nanocrystal surface destabilization from Cl^–^ removal is likely the driving force for particle aggregation.

As oxidation with MX_2_/TOP provides postsynthetic access to highly Cu-deficient Cu_3–*x*_P, this chemistry could enable further study of the electrochemical lithiation mechanism for *P*6_3_*cm* Cu_3–*x*_P. The electrochemical lithiation of bulk Cu_3–*x*_P is known to extrude crystalline Cu(0) through (partial) substitution reactions that accompany the formation of ternary phases Li_2_CuP and Li_3_P with increasing negative potential.^78^ The initial discharge step is controversial, with conflicting literature accounts of whether or not monophasic Li intercalation is possible without cation exchange^78,79^ at less negative potentials prior to formation of Li_2_CuP. Recent work from Wolff and co-workers has suggested that initially Cu-deficient *P*6_3_*cm* Cu_3–*x*_P undergoes a different lithiation pathway than the more Cu-rich counterpart, as indicated by differential capacity measurements.^1^ Their interpretation of this result was that the Cu-deficient composition does not extrude Cu(0) prior to reaching the potential at which Li_2_CuP begins to form. The MX_2_/TOP oxidation chemistry of this study, which, to our knowledge, yields the smallest *P*6_3_*cm* Cu_3–*x*_P unit-cell volume reported in the literature, allows for access of a highly Cu-deficient Cu_3–*x*_P composition. Accordingly, the Cu_3–*x*_P nanocrystal thin films oxidized with MX_2_/TOP would be ideal candidates for fundamental electrochemical lithiation studies. If monophasic, reductive Li^+^ intercalation without Cu(0) extrusion is indeed possible for sufficiently copper-deficient *P*6_3_*cm* Cu_3–*x*_P films, it could be used to access rapid and reversible plasmonic tunability while also avoiding the aforementioned limitations of solution-phase redox chemistry.

## Summary and Conclusions

We have developed three postsynthetic chemistries for the anaerobic Cu-coupled redox modulation of plasmonic Cu_3–*x*_P nanocrystals. These redox chemistries yield correlated changes to the optical, electronic, and structural properties of Cu_3–*x*_P nanocrystals. Similar to copper chalcogenides, postsynthetic reactions with Cu^+^ and I_2_ lead to nanocrystal reduction and oxidation, respectively. This redox activity is demonstrated through observation of a shift in the LSPR energy, a change in the lattice parameters, and a Knight shift in the solid-state ^31^P MAS NMR spectra. A previously unreported Cu-coupled oxidation chemistry with MX_2_/TOP accesses a greater range of structural and electronic properties of *P*6_3_*cm* Cu_3–*x*_P than has been previously achieved. Cu_3–*x*_P nanocrystals oxidized with MX_2_/TOP can be back-reduced with Cu^+^, indicating that this oxidation chemistry permits reversible carrier density tuning and does not irrevocably change the material. Additionally, control experiments were used to determine that MX_2_ identity is generalizable to zinc and cadmium halide salts and that MX_2_ and TOP are both needed for the full oxidative effect. The three redox chemistries and corresponding characterization presented herein will inform future endeavors to leverage the optical, structural, and compositional tunability of Cu_3–*x*_P nanocrystals.

## Experimental Methods

### Chemicals

The manufacturers and purities of the chemicals used in this study are given in Table S10. Toluene, tetrahydrofuran (THF), and acetonitrile (MeCN) were dried and deoxygenated according to standard procedures^80^ and stored over molecular sieves (3 Å) for 2 days before use. Methanol (MeOH) was deoxygenated with several freeze–pump–thaw cycles and stored over molecular sieves (3 Å) for 2 days before use. Oleylamine (OAm), trioctylamine (TOA), and tris(diethylamino)phosphine (P(NEt_2_)_3_) were degassed upon receipt and stored over molecular sieves (4 Å) for 2 days before use. All zinc and cadmium salts were dried under vacuum at 120 °C for 2 h before use. Nitric acid and copper metal were used as received and handled in air. All other chemicals were handled and stored exclusively in a nitrogen-filled glovebox. Lithium metal was opened in a nitrogen-filled glovebox and promptly transferred to rubber-septum-capped vials, which were evacuated and stored under static vacuum to limit the formation of lithium nitride.

### Synthesis

Synthesis and workup of Cu_3–*x*_P nanocrystals were conducted under inert atmosphere to avoid aerobic oxidation, either with a nitrogen-equipped Schlenk line or in a nitrogen-filled glovebox.

#### One-Pot Syntheses of Cu_3–*x*_P Nanocrystals with TOA as a Cosolvent

The synthesis of Cu_3–*x*_P nanocrystals was adapted from a previous study.^16^ For the syntheses reported herein, the reaction mixture compositions were identical within the measurement limitations (Table S1). For Syntheses NS1 and NS2, the temperature profiles and reaction times were different than those of Syntheses 1–8.

In a typical synthesis (Syntheses 1–8, NS1), a three-neck, 25 mL round-bottom flask was loaded with CuCl (79.2 mg, 0.800 mmol), P(NEt_2_)_3_ (277 mg, 1.12 mmol), OAm (1.05 g, 1.29 mL, 3.93 mmol), and TOA (5.43 g, 6.71 mL). This gave a total volume of 8.00 mL with OAm/P/Cu = 4.9/1.4/1.0 and [Cu] = 0.100 M. The flask was equipped with a condenser and two septa through which glass sheaths containing 2 cm silicone oil were inserted. To the sheaths were added a thermocouple to control the temperature and an Omega data logger to measure the temperature. The mixture was stirred and degassed at ∼125 °C for 3 h. At this point, the pressure over the flask was equal to the equilibrium, closed-vacuum-line pressure, indicating full removal of HNEt_2_ that was generated by transamination of P(NEt_2_)_3_. The resulting pale, straw-colored solution was placed under nitrogen and was heated as outlined in Heating Methods after first equilibrating to a starting temperature of 105 °C. Following the reaction, the heating mantle was removed and the flask allowed to slowly cool. The room-temperature reaction flask was transferred to a nitrogen-filled glovebox. In the flask were placed 15 mL THF and 7 mL MeCN. The resulting suspension was transferred to two test tubes, and the nanocrystals were collected via centrifugation at 2000 rpm for 5 min. The nanocrystals were resuspended in 3000 μL of toluene to form concentrated stock solutions.

For Synthesis NS2, the reaction conditions were the same with the exception of a few changes made to account for the smaller scale and lower total volume of 4.97 mL. The flask was loaded with CuCl (49.1 mg, 0.496 mmol), P(NEt_2_)_3_ (173 mg, 0.699 mmol), OAm (0.662 g, 0.814 mL, 2.47 mmol), and TOA (3.35 g, 4.14 mL). 10 mL THF and 6 mL MeCN were added to the mixture prior to centrifugation, and the nanocrystals were resuspended in 2000 μL of toluene after collection by centrifugation. The ensemble from Synthesis NS2 was used for a single oxidation-with-I_2_ experiment solely for the purpose of verifying CuI formation by powder X-ray diffraction (Figure S9). Characterization of the Synthesis NS2 ensemble by TEM, absorption spectroscopy, and powder X-ray diffraction (Figure S37) reveals Cu_3–*x*_P nanocrystals similar to those isolated from Syntheses 1–8, despite the altered temperature profile of the former from its lower reaction volume.

#### Heating Methods

For Syntheses 1–8 and NS2, the reaction temperature during degassing was controlled with a 25 mL heating mantle connected to a proportional–integral–derivative controller. After the degassing step, the heating mantle was instead controlled with a contact voltage regulator (Volteq, TDGC_2_–500VA). The desired heating profile was achieved through a stepwise, two-voltage scheme. For Syntheses 1–8, a starting voltage of 120 V was used to set the maximum ramp rate (d*T*/d*t* ≈ 22 °C/min) with a starting temperature of 105 °C. When the temperature reached ∼230 °C after 7 min, the voltage was lowered to 90 V to set the asymptotic temperature of ∼272 °C for the remaining 10 min of the reaction. This two-step constant voltage heading method led to reproducible temperature profiles across multiple syntheses (Figure S1). For Synthesis NS2, a starting voltage of 120 V was used to set the maximum ramp rate (d*T*/d*t* = 35 °C/min) with a starting temperature of 115 °C. When the temperature reached 205 °C after 3.5 min, the voltage was lowered to 90 V for the remaining 13 min of the reaction to reach *T*_f_ = 276 °C (Figure S37a). The lower reaction volume of 4.96 mL for Synthesis NS2 (Table S1) was the cause for the different temperature profile as compared to Syntheses 1–8. For Synthesis NS1 (low temperature), the heating mantle was entirely controlled with a PID. Following degassing, the reaction mixture was heated (maximum ramp rate d*T*/d*t* ≈ 19 °C/min) to *T*_f_ = 200 °C with an initial temperature overshoot of *T*_max_ = 215 °C (Figure S26) and held for a total reaction time of 21 h.

#### Quantification of the Reaction Temperature

The reaction temperature was stored digitally with an Omega data logger (OM-EL-USB-TC-LCD) at 5 s intervals for Syntheses 1–8 and NS2. The reaction temperature was recorded by hand at 60 s intervals for Synthesis NS1. The raw temperature was adjusted to a corrected temperature with a temperature-dependent empirical quadratic function which was generated by calibration against an external mercury thermometer.

### Postsynthetic Redox Treatments

All postsynthetic redox treatments of Cu_3–*x*_P nanocrystals were conducted under an inert atmosphere to avoid aerobic oxidation, either with a nitrogen-equipped Schlenk line or in a nitrogen-filled glovebox. After each postsynthetic redox treatment, nanocrystals were resuspended in toluene without additional washes to avoid aggregation. Subsequent washes were performed immediately prior to characterization and are described in the corresponding sections.

#### Oxidation of Cu_3–*x*_P Nanocrystals with I_2_

In a nitrogen-filled glovebox, a three-neck, 25 mL round-bottom flask was loaded with a stock solution of Cu_3–*x*_P nanocrystals (from Synthesis 2; 2.97 mL, 0.65 mmol Cu). The flask was equipped with a reflux condenser and two rubber septa and transferred to a Schlenk line. Under high nitrogen flow, the rubber septum on one side neck was replaced with a Merlic solid-addition adapter loaded with I_2_ (5.0 g, 20 mmol). With stirring, the flask was subjected to ten cycles of 5 s dynamic vacuum followed by 55 s static vacuum. The reaction was left under static vacuum for 20 h with vigorous stirring. This reaction setup, which maintained physical separation of the solid I_2_ from the nanocrystal suspension, is shown in Figure S4. The oxidation was terminated by replacement of the iodine-filled adapter with a rubber septum under a high nitrogen flow. The remaining volatiles were removed from the flask under vacuum for 30 min. The flask was transferred back to the glovebox, where nanocrystals were resuspended in 2970 μL of toluene to form a concentrated stock solution. The washing procedures for subsequent characterizations are described in the corresponding sections. All supernatants from these washes were combined and concentrated under vacuum to give a waxy, white residue. All of the residue was digested in 2000 μL of nitric acid to determine the Cu content by electronic absorption spectroscopy.

For the oxidation-with-I_2_ experiment using the ensemble from Synthesis NS2, the reaction conditions were the same with a few differences. The stock solution of Cu_3–*x*_P nanocrystals used had lower volume and lower concentration (1.00 mL, 0.18 mmol Cu). After the oxidation, the nanocrystals were resuspended in 1000 μL of toluene. Without the addition of MeCN, the toluene suspension was transferred to a single, counterbalanced test tube and a white solid was collected via centrifugation at 2000 rpm for 1 min. This white solid was characterized by powder X-ray diffraction and found to contain CuI.

#### Reduction of Cu_3–*x*_P Nanocrystals with Cu^+^

The procedure to reduce Cu_3*–x*_P nanocrystals with Cu^+^ was adapted from a previous report.^31^ In a nitrogen-filled glovebox, a 20 mL scintillation vial was loaded with Cu_3–*x*_P nanocrystals in a toluene suspension (2.97 mL, 0.64 mmol Cu), THF (15 mL), OAm (100 μL, 0.3 mmol), [Cu(MeCN)_4_]PF_6_ (56 mg, 0.15 mmol), and MeOH (200 μL, 4.94 mmol). The mixture was vigorously stirred for 20 h at ambient temperature. The reduction was terminated by the addition of 800 μL of OAm (to prevent aggregation) and 2 mL MeCN (to precipitate nanocrystals) to the mixture. The nanocrystals were transferred to two test tubes, collected via centrifugation at 2000 rpm for 5 min, and resuspended in 2970 μL of toluene. The washing procedures for subsequent characterizations are described in the corresponding sections. All supernatants from these washes were added to the supernatant collected from this initial wash. The combined supernatant was concentrated under vacuum to yield an oil, which was analyzed by electronic absorption and solution NMR spectroscopies.

For the experiment using the Synthesis NS1 ensemble, the scale was reduced to ∼30% with otherwise identical conditions. A 20 mL scintillation vial was loaded with Cu_3–*x*_P nanocrystals in a toluene suspension (900 μL, 0.20 mmol Cu), THF (4.5 mL), OAm (30 μL, 0.09 mmol), [Cu(MeCN)_4_]PF_6_ (17 mg, 0.047 mmol), and MeOH (60 μL, 1.5 mmol). The reduction was terminated with only 300 μL of OAm and 600 mL MeCN. The nanocrystals were resuspended in 900 μL of toluene.

#### Oxidation of Cu_3–*x*_P Nanocrystals with MX_2_/TOP (M = Zn, Cd; X = Cl, I, Acetate)

For the standard ZnI_2_/TOP condition (ZnI_2_/TOP/Cu = 3.8/15/1), in a nitrogen-filled glovebox, a 20 mL scintillation vial was loaded with Cu_3–*x*_P nanocrystals in a toluene suspension (from Synthesis 4; 2.97 mL, 0.67 mmol Cu), ZnI_2_ (0.809 g, 2.53 mmol), TOP (3.76 g, 4.52 mL, 10.1 mmol), and OAm (0.508 g, 0.63 mL, 1.90 mmol). The vial was sealed with a Teflon-lined cap and heated to 100 °C with vigorous stirring for 20 h. The oxidation was terminated by the addition of 12 mL THF (for miscibility) and 6 mL MeCN (for precipitation) to the mixture. The slurry was transferred to two test tubes, and nanocrystals were collected via centrifugation at 2000 rpm for 5 min and resuspended in 2970 μL of toluene. The washing procedures for subsequent characterizations are described in the corresponding sections. All supernatants from these washes were added to the supernatant collected from this initial wash. The combined supernatant was concentrated under vacuum to yield an oil, which was analyzed by electronic absorption and solution NMR spectroscopies.

For the experiment using the Synthesis NS1 ensemble, the scale was reduced to ∼30% with otherwise identical conditions. A 20 mL scintillation vial was loaded with Cu_3–*x*_P nanocrystals in a toluene suspension (900 μL, 0.20 mmol Cu), ZnI_2_ (0.245 g, 0.767 mmol), TOP (1.14 g, 1.37 mL, 3.08 mmol), and OAm (0.16 g, 0.20 mL, 0.60 mmol). The oxidation was terminated with only 4 mL THF and 2 mL MeCN. The nanocrystals were resuspended in 900 μL of toluene.

For the remaining experiments using the Synthesis 7 ensemble (corresponding to Figures 5, Figures S28 and S29), the most important differences in the reaction conditions involved changing the identity or concentration of MX_2_ or omitting TOP and/or MX_2_, with use of additional toluene to maintain [Cu] = 0.081 M (Table S6). These 9 oxidation reactions were carried out in parallel to ensure consistent heating among the experiments. Another substantial difference is that the MX_2_/TOP/OAm/toluene mixtures were first heated to 100 °C for 3 h in the absence of Cu_3–*x*_P nanocrystals to verify solubilization of the MX_2_ salt prior to the oxidation. A toluene suspension of Cu_3–*x*_P nanocrystals (300 μL, 0.066 mmol of Cu) was added to bring the solution to [Cu] = 0.081 M, which was then heated for an additional 20 h. The oxidations were each terminated with only 3 mL THF and 1.5 mL MeCN. The nanocrystal ensembles from the oxidation experiments were each separately resuspended in 300 μL of toluene.

#### Treatment of Cu_3–*x*_P Nanocrystals with Li

In a nitrogen-filled glovebox, a 20 mL scintillation vial was loaded with Cu_3–*x*_P nanocrystals in a toluene suspension (from Synthesis 5; 2.97 mL, 0.62 mmol Cu), THF (0.33 mL, 4.1 mmol), and Li^0^ (0.15 g, 22 mmol). The mixture was vigorously stirred at ambient temperature for 20 h and monitored via electronic absorption spectroscopy. After 20 h, when it became necessary to reconstitute colloidal stability Cu_3–*x*_P nanocrystals for absorption spectroscopy, the aggregated suspension was decanted to a vial separate from the Li^0^ granules. For electronic absorption measurements, colloidal stability was reconstituted through the addition of the nanocrystal mixture to OAm-containing solutions of THF/toluene (0.4% OAm, 49.8% THF, 49.8% toluene by volume in the final suspension). Sample preparation for powder X-ray diffraction and solid-state NMR was performed on the same day to avoid reversal of lithiation by minimizing postredox time in the solution state.

#### Treatment of Cu_3–*x*_P Nanocrystals with Li in the Presence of Excess OAm

In a nitrogen-filled glovebox, a 20 mL scintillation vial was loaded with Cu_3–*x*_P nanocrystals in a toluene suspension (from Synthesis 6; 600 μL, 0.13 mmol Cu), OAm (66 μL, 0.20 mmol), and Li (30 mg, 4.3 mmol). The mixture was vigorously stirred at ambient temperature, and it appeared colloidally stable throughout the reaction. The reduction was terminated after 96 h by decanting the colloidally stable Cu_3*–x*_P nanocrystal suspension to a vial separate from the Li granules. The nanocrystal suspension was diluted with 2 mL toluene and 10 mL THF (for miscibility) and 8 mL MeCN (for precipitation), and the nanocrystals were subsequently precipitated with the addition of 8 mL MeCN. The slurry was transferred to a test tube, and nanocrystals were collected via centrifugation at 2000 rpm for 5 min. The nanocrystals were resuspended in 600 μL of toluene. Absorption spectroscopy was performed immediately upon resuspension of the nanocrystals, as the observed reduction was easily reversible. The product from this same experiment was used for a reduction reversibility experiment with I_2_ (see below).

#### Redox Reversibility Experiments

The reversibility experiments were performed on a reduced scale with use of 600 μL of Cu_3–*x*_P nanocrystal stock solution (0.13 mmol Cu) for each experiment to allow use of the same ensemble (from Synthesis 6) for all of them. The individual redox experiments were performed as noted above, and identical components of the protocols are not repeated. The following is an example of how two redox experiments were performed sequentially, using the case of the oxidation-with-ZnI_2_/TOP reversibility experiment (Figure 4). A 20 mL scintillation vial was loaded with Cu_3–*x*_P nanocrystals in a toluene suspension (600 μL, 0.13 mmol Cu), ZnI_2_ (0.164 g, 0.514 mmol), TOP (0.745 g, 0.897 mL, 2.01 mmol), and OAm (0.118 g, 0.145 mL, 0.441 mmol). After the oxidation, 3 mL THF and 1.5 mL MeCN were added to the mixture. The slurry was transferred to two test tubes, and nanocrystals were collected via centrifugation at 2000 rpm for 5 min and resuspended in 600 μL of toluene. Half of the resulting stock solution (300 μL) was kept for TEM, absorbance, and powder X-ray diffraction characterization of the midpoint product. The remaining 300 μL was used for the reduction reversibility with Cu^+^. A 20 mL scintillation vial was loaded with 300 μL of stock solution of Cu_3–*x*_P nanocrystals, THF (1.5 mL), OAm (10 μL, 0.03 mmol), [Cu(MeCN)_4_]PF_6_ (6.1 mg, 0.016 mmol), and MeOH (20 μL, 0.49 mmol). The reduction was terminated by the addition of 80 μL of OAm and 200 μL of MeCN to the mixture. The nanocrystals were transferred to two test tubes, collected via centrifugation at 2000 rpm for 5 min, and resuspended in 300 μL of toluene.

Similar to the oxidation-with-ZnI_2_/TOP reversibility experiment, the remaining 3 reversibility experiments were conducted at 20% scale (600 μL of stock solution) for the initial redox treatment and 10% scale (300 μL of stock solution) for the subsequent reversibility experiment with a single centrifugation–purification step between the two experiments.

#### Synthesis and Isolation of Ferrocenium Triflate (FcOTf)

In a nitrogen-filled glovebox, a 20 mL scintillation vial was loaded with silver triflate (0.205 g, 0.778 mmol), ferrocene (0.148 g, 0.796 mmol), THF (3 mL), and MeCN (1 mL, 19 mmol). The solution was vigorously stirred at ambient temperature for 12 h and the metallic silver was filtered off. The dark blue solution was concentrated under vacuum to give FcOTf as a dark blue powder (0.247 g, 94.7% yield).

#### Cuvette-Scale Oxidation of Cu_3–*x*_P Nanocrystals with Cerium(IV) Ammonium Nitrate (CAN)

In a nitrogen-filled glovebox, a toluene suspension of Cu_3–*x*_P nanocrystals (from Synthesis 6; 10 μL, 2.1 μmol Cu), THF (400 μL) and toluene (390 μL) were added to a 2 mm quartz screw-cap cuvette. The absorption was monitored prior to redox and after each successive addition of 3 μL of a MeOH solution of CAN (0.101 M) up to a total of 9 μL (Figure S16ai). The full oxidation corresponds to 0.43 CAN/Cu. Once the oxidation was complete, 400 μL of MeCN was added, and the slurry was transferred to a plastic, O-ring, screw-cap tube (Axygen, SCT-200-SS-C). The nanocrystals were collected via centrifugation at 4000 rpm for 5 min and subsequently resuspended in 800 μL of 1/1 toluene/THF (by volume) for TEM imaging.

#### Cuvette-Scale Oxidation of Cu_3–*x*_P Nanocrystals with FcOTf and Bu_4_NI

In a nitrogen-filled glovebox, a toluene suspension of Cu_3–*x*_P nanocrystals (from Synthesis 6; 10 μL, 2.1 μmol Cu), THF (400 μL), and toluene (390 μL) were added to a 2 mm quartz screw-cap cuvette. The absorption was monitored prior to redox and after each successive addition of 3 μL of both (1) a MeCN solution of tetrabutylammonium iodide (Bu_4_NI,0.066 M) and (2) a MeCN solution of FcOTf (0.059 M) up to a total of 9 μL each (Figure S16bi). The full oxidation corresponded to 0.28 TBA/Cu and 0.25 Fc/Cu. Once the oxidation was complete, 400 μL of MeCN was added, and the slurry was transferred to a plastic, O-ring, screw-cap tube (Axygen, SCT-200-SS-C). The nanocrystals were collected via centrifugation at 4000 rpm for 5 min and subsequently resuspended in 800 μL of 1/1 toluene/THF (by volume) for TEM imaging.

### Characterization

All sample preparations for characterization were conducted under an inert atmosphere in a nitrogen-filled glovebox to mitigate aerobic oxidation prior to measurement.

#### TEM

Sample grid preparation involved preparing a dilute Cu_3–*x*_P nanocrystal solution by the addition of 10 μL of toluene stock solution (∼2.1 μmol Cu) to 790 μL of toluene/THF (39/40 by volume). 10 μL of this dilute Cu_3–*x*_P nanocrystal solution was drop-cast onto a 100 mesh copper grid coated with Formvar and carbon (Electron Microscopy Sciences). The copper grid was suspended in air with reverse action tweezers to allow the entire 10 μL droplet to evaporate directly from the grid. Imaging was performed on the same day as grid preparation to avoid particle oxidation. Images were collected on a FEI Tecnai Spirit operating at 120 kV. The lateral dimension was taken to be the longest line that could be drawn along the two-dimensional projection of a nanoplatelet. The size distributions were derived from the analysis of 150 nanocrystals with the use of 13(√150 + 1) constant-width bins spanning the ensemble range.

#### Electronic Absorption Spectroscopy of Cu_3–*x*_P Nanocrystals

In a nitrogen-filled glovebox, a 2 mm quartz screw-cap cuvette (Starna Cells 18F-Q-10-Gl14-C) was loaded with Cu_3–*x*_P nanocrystals in a toluene suspension (10 μL, ∼2.1 μmol Cu), 390 μL of toluene, and 400 μL of THF. Spectra were collected using a Cary 5000 spectrometer in transmission mode. A background spectrum of 1/1 THF/toluene (by volume) in the same cuvette was subtracted from each spectrum. The LSPR energies were estimated by local fitting of the near-IR absorbance peak to a Gaussian function with use of a linear background to account for nonplasmonic near-IR baseline absorption.

#### Powder X-ray Diffraction

From the concentrated toluene stock solution, 300 μL (∼0.064 mmol Cu) was transferred to a plastic, O-ring, screw-cap tube (Axygen, SCT-200-SS-C) and precipitated with 300 μL of MeCN. The precipitated nanocrystals were collected via centrifugation at 4000 rpm for 5 min. The nanocrystals were resuspended in 300 μL of THF, precipitated with 300 μL of MeCN, and collected via centrifugation at 4000 rpm for 5 min. Residual solvent was removed under vacuum, and the pellets (∼5 mg) were stored under nitrogen until just before the measurement.

Powder diffraction patterns were measured using a transmission-mode diffractometer equipped with a Bruker Apex II detector and Cu Kα radiation source (λ = 1.5418 Å). In a typical measurement, a 200 μm diameter piece of pellet was quickly picked out under a light microscope and mounted onto the diffractometer goniometer head and kept under a dry nitrogen stream (*T* = 280 K; 5 L/min flow rate) to minimize air exposure. For the as-synthesized Synthesis 1 ensemble and postredox Syntheses 2–5 ensembles, samples were measured in triplicate, with different pellet pieces each time. Three frames centered at 2θ = 30, 45, and 60°, respectively, were collected with a detector distance of 150 mm, spinning φ, and 240 s exposure time per image. The Debye rings obtained from the three frames were merged and radially integrated in a cone with γ range 80–100° using Diffrac.eva software. The resulting powder patterns were fit in the range of 2θ = 20–70° with a cubic baseline and Gaussian peaks using the Wavemetrics Multi-Peak Fitting Package in Igor Pro 6.37. An external standard of bulk lanthanum hexaboride was used to correct systematic errors in 2θ. For comparison, the powder diffraction pattern of Cu_2.87_P was simulated from single-crystal data^12^ using VESTA.^81^ Platelet lateral dimensions were estimated for each ensemble through Scherrer analyses of the (300) reflection, as it has no contribution from the *c*-direction. These values were then used to determine the nanoplatelet height using Scherrer analysis of the (112) reflection.

#### Digestion of Cu_3–*x*_P Nanocrystals for Determination of Cu-Content

In a nitrogen-filled glovebox, 50 μL of stock solution (∼0.011 mmol Cu) was transferred to a plastic, O-ring, screw-cap tube (Axygen, SCT-200-SS-C), diluted with 300 μL of THF, and precipitated with 600 μL of MeCN. The precipitated nanocrystals were collected via centrifugation at 4000 rpm for 5 min. The nanocrystals were resuspended in 300 μL of THF, precipitated with 600 μL of MeCN, and collected via centrifugation at 4000 rpm for 5 min. This process of withdrawing and washing 50 μL of the stock solution was repeated a total of three times to give three separate pellets. The pellets were left in air to allow residual solvent evaporation, after which they were individually digested overnight in the sealed O-ring, screw-cap tube with 800 μL of 70% HNO_3_ (Fisher Chemical, certified ACS plus). The resulting solutions were filtered with a 0.22 μm PVDF syringe filter and transferred to a 2 mm path length cuvette. Absorption spectra were collected using a Cary 5000 spectrometer in transmission mode. A background of 70% HNO_3_ was subtracted from each spectrum. To determine [Cu^2+^], the extinction coefficient under these conditions was determined by generating a calibration line using analogously digested Cu (Figure S3b). Rather than using a single-energy extinction coefficient for [Cu^2+^] quantification, an integrated energy range of 1.378–1.550 eV was used. It was assumed that all Cu from the digested Cu_3–*x*_P nanocrystals were oxidized to Cu^2+^.

#### Determination of Synthesis Yields

The Cu contents of Cu_3–*x*_P nanocrystals from Syntheses 1 and NS1 were determined as described above and used to determine the yields based on Cu for those syntheses. For other syntheses, the yield was estimated using a Cu-based extinction coefficient at 3.1 eV of *ε*_3.1 eV_^Cu^ = 2100 ± 200 L/((mol Cu) cm). This extinction coefficient was derived from the copper content and absorption spectra of Cu_3–*x*_P nanocrystals from Synthesis 1.

#### Solid-State ^31^P MAS NMR Spectroscopy

In a nitrogen-filled glovebox, 2400 μL (∼0.51 mmol Cu) stock solution was transferred to a test tube and precipitated with 1500 μL of MeCN. The precipitated nanocrystals were collected via centrifugation at 2000 rpm for 5 min. The resulting pellet was resuspended in 2000 μL of toluene, precipitated with 1000 μL of MeCN and collected by centrifugation at 2000 rpm for 5 min. The resulting pellet was dried under vacuum, pulverized, and stored under static vacuum in rubber-septum-capped test tubes until the measurement. In a typical workup, the pellet mass was ∼40–50 mg. The pellet of nanocrystals reduced with Cu^+^, however, was 86 mg.

All ^31^P MAS NMR experiments were performed with a 600 MHz (14.1 T) Bruker AVANCE-IIIHD spectrometer equipped with a Bruker 3.2 mm HPC MAS probe. In a nitrogen-filled glovebox, 3.2 mm rotors were center packed with Teflon tape and nanocrystalline Cu_3–*x*_P powder in a glovebox. ^31^P direct polarization experiments were conducted with 12 kHz MAS and collected with 128 scans. The π/2 pulse length was 2 μs, and the recycle delay was 3 s. ^31^P *T*_1_ values were determined using saturation recovery experiments with 12 kHz MAS and collected with 128 scans with variable delay times from 0.0005 to 1.0 s. ^31^P chemical shifts were referenced externally to Ca_3_(PO_4_)_2_ (upfield resonance at −1.66 ppm relative to 85% phosphoric acid).

#### Elemental Analysis by ICP–MS

The same solid pellets used for powder X-ray diffraction were subsequently used for ICP–MS. The remainder of the pellets was digested overnight in a sealed plastic, O-ring, screw-cap tube (Axygen, SCT-200-SS-C) with 800 μL of 70% HNO_3_ (Fisher Chemical, TraceMetal). The following day, the solutions were filtered with a 0.22 μm PVDF syringe filter, diluted with 5/95 HNO_3_/ultrapure water (by volume), and analyzed using a Thermo iCAP RQ ICP–MS. The ultrapure water was collected from a Barnstead Nanopure Water Purification System (Thermo Fisher). Serial dilution was used to prepare three solutions for each sample with cumulative dilution factors of 4167, 8333, and 16667. For each sample, element quantification is reported as an average for all values which fall within the calibration range.

#### Solution NMR Spectroscopy

In a typical preparation, 100 μL of the supernatant and 600 μL of benzene-*d*_6_ were added to a screw-cap NMR tube (Wilmad-Labglass). ^31^P spectra were collected on a Varian Mercury Plus 400 MHz spectrometer (162 MHz) or a JEOL ECA 500 MHz spectrometer (203 MHz) with ^1^H decoupling. ^31^P NMR spectra of solutions containing TOP were collected with 1024 scans with relaxation delay 0.5 s and processed in Mestrenova using 2 Hz apodization. ^31^P NMR spectra of solutions containing [PF_6_]^−^ were collected with 64 scans and relaxation delay 1 s and processed using 5 Hz apodization. ^19^F spectra were collected on a JEOL ECA 500 MHz spectrometer (476 MHz) with 16 scans and relaxation delay 1 s and processed using 5 Hz apodization. Chemical shifts for ^31^P NMR spectra were externally referenced to 85% H_3_PO_4_ (δ = 0 ppm).

#### STEM–EDS

Sample grid preparation involved preparing a dilute Cu_3–*x*_P nanocrystal solution by the addition of 10 μL of toluene stock solution (∼2.1 μmol Cu) to 790 μL of toluene. 10 μL of this dilute Cu_3–*x*_P nanocrystal solution was drop-cast onto a 400 mesh gold grid coated with lacey carbon and without Formvar (Ted Pella, Inc.). The gold grid was suspended in air with reverse action tweezers to allow the entire 10 μL droplet to evaporate directly from the grid. STEM images and spatially correlated EDS spectra were collected on a JEM-ARM300F Grand Arm instrument operating at 300 kV. STEM images, EDS color maps, and intensity integration area scans were processed with Gatan DigitalMicrograph software. 3D EDS surface plots were generated in MATLAB after processing the EDS color map images with pixel-wise adaptive Wiener noise-removal filtering (the “wiener2” function from the Image Processing Toolbox).

## Supporting Information Available

Additional details on synthetic conditions of each synthesis, workflow schemes, tabulated data, additional absorption, TEM, diffraction, and NMR characterization of Cu_3–*x*_P nanocrystals before and after redox treatments, characterization of additional redox treatments, data and analysis used for determining synthesis and postredox yields, data and analysis of supernatants/byproducts of redox treatments, description of absorption analysis, characterization of additional reversibility experiments, STEM–EDS mapping images, literature data used for determining empirical relationships, and TEM characterization of aerobically oxidized Cu_3–*x*_P nanocrystals (PDF)
