# Supplementary material for: Redox Chemistries for Vacancy Modulation in Plasmonic Copper Phosphide Nanocrystals
Source: ACS Nano. 2024 Feb 7;18(7):5282–96. doi: 10.1021/acsnano.3c08962 (PMC10883034; doi:10.1021/acsnano.3c08962)
Supplement: Supplementary file 1 — nn3c08962_si_001.pdf [file nn3c08962_si_001.pdf]

Supporting Information for

Redox Chemistries for Vacancy Modulation in Plasmonic Copper Phosphide Nanocrystals

Alexander G. Rachkov,[1] Kevin Chalek,[2] Hang Yin,[1] Mingjie Xu,[3] Gregory P. Holland,[2] and Alina M. Schimpf[1,4]*

[1]*Department of Chemistry and Biochemistry, University of California, San Diego, La Jolla, CA 92093, USA*
[2]*Department of Chemistry and Biochemistry, San Diego State University, San Diego, CA 92182, USA*
[3]*Irvine Materials Research Institute (IMRI) University of California, Irvine, CA 92697, USA*
[4]*Program in Materials Science and Engineering, University of California, San Diego, La Jolla, CA 92093, USA*
**Electronic address: aschimpf@ucsd.edu*

Supplementary Tables, Figures and Analysis 3
Table S1. Details of all $Cu_{3-x}P$ nanocrystal syntheses 3
Figure S1. Temperature profiles of standard $Cu_{3-x}P$ nanocrystal syntheses 4
Figure S2. Three absorption spectra of $Cu_{3-x}P$ nanocrystals from Synthesis 1 4
Figure S3. Quantification of Cu-content by digestion 5
Scheme S1. Workflow for redox treatments 6
Figure S4. Photographs of oxidation with I_2 6
Table S2. Post-redox Cu-content and LSPR shifts 7
Figure S5. TEM characterization of nanocrystals before and after redox treatments 8
Figure S6. Powder X-ray diffraction patterns of nanocrystals after redox treatments 9
Figure S7. Absorption spectra of digested $Cu_{3-x}P$ nanocrystals after redox treatments 9
Section S1. Analysis of the supernatant following oxidation with I_2 10
Figure S8. Analysis of the supernatant following oxidation with I_2 11
Figure S9. Powder X-ray diffraction of supernatant residue following oxidation with I_2 11
Figure S10. Analysis of the supernatant following reduction with Cu^+ 12
Section S2. Analysis of the supernatant following reduction with Cu^+ 13
Figure S11. Optical analysis of Cu^+ co-precipitation control experiment 14
Figure S12. Aerobic absorbance of supernatant following oxidation with ZnI_2 and TOP 14
Section S3. Analysis of the supernatant following oxidation with ZnI_2 and TOP 15
Figure S13. ^{31}P-NMR analysis of supernatant following oxidation with ZnI_2/TOP 16
Figure S14. Characterization of Li-treated nanocrystals 17
Figure S15. Absorption spectra of nanocrystals reduced with Li in excess OAm 18
Figure S16. Characterization of $Cu_{3-x}P$ nanocrystals treated with other oxidants 18
Figure S17. Unit-cell parameters as a function of LSPR energy 19
Figure S18. Unit-cell volume as a function of composition for bulk $Cu_{3-x}P$ 19
Section S4. Drude–Lorentz modeling of optical data 20
Figure S19. Saturation–recovery experiments of as-synthesized $Cu_{3-x}P$ nanocrystals 21
Figure S20. Delocalized hole concentration vs Cu vacancy concentration 21
Table S3. Relationship between unit-cell volume and ^{31}P chemical shift 21
Figure S21. Saturation–recovery experiments of redox-treated $Cu_{3-x}P$ nanocrystals 22
Scheme S2. Workflow of redox reversibility experiments. 23

Figure S22. TEM and X-ray diffraction for reversibility of oxidation with ZnI_2/TOP..........23
Table S4. Collected compositional, optical and structural data..........24
Table S5. Scherrer analysis of nanocrystal sizes in reversibility experiments..........25
Figure S23. Characterization for the reversibility of oxidation with I_2..........25
Figure S24. Characterization for the reversibility of reduction with Cu^+..........26
Figure S25. Characterization for the reduction reversibility experiment with Li/OAm..........27
Figure S26. Synthesis and redox treatments of Cu-deficient $Cu_{3-x}P$ nanocrystals..........28
Figure S27. TEM of Cu-deficient $Cu_{3-x}P$ nanocrystals..........28
Table S6. Details of post-synthetic MX_2/TOP experiments..........29
Figure S28. TEM characterization of MX_2/TOP treatments..........30
Figure S29. Powder X-ray diffraction following various MX_2/TOP treatments..........31
Table S7. ICP–MS elemental analysis of select $Cu_{3-x}P$ nanocrystal samples..........32
Figure S30. STEM–EDS mapping images of as-synthesized $Cu_{3-x}P$ nanocrystals..........32
Figure S31. STEM–EDS mapping images of nanocrystals oxidized with ZnI_2/TOP..........33
Figure S32. STEM–EDS mapping images of nanocrystals oxidized with ZnI_2/TOP..........33
Figure S33. STEM–EDS mapping images of nanocrystals oxidized with CdI_2/TOP..........34
Figure S34. STEM–EDS mapping images of nanocrystals oxidized with CdI_2/TOP..........35
Figure S35. Aerobic surface oxidation of $Cu_{3-x}P$ nanocrystals..........36
Table S8. $Cu_{3-x}P$ nanocrystal lateral platelet size from TEM and Scherrer analysis..........37
Figure S36. E_{LSPR} and (300) as a function of Cu/P..........38
Table S9. Correlation between ΔCu and nanocrystal properties..........38
Table S10. Chemicals..........39
Figure S37. Nonstandard synthesis of $Cu_{3-x}P$ nanocrystals (Synthesis NS2)..........40

References..........41

Supplementary Tables, Figures and Analysis

Table S1. Synthetic details and characterization of all $Cu_{3-x}P$ nanocrystal syntheses used herein. All syntheses had [Cu] = 0.100 M.

Synthesis	OAm/P/Cu	Volume (ml)	T_f (°C)	dT/dt (°C/min)	% Yield (Cu)[a]	LSPR Max (meV)	TEM Lateral Size (nm)[b]	Primary Figures
Standard Conditions								
**1**	4.91/1.40/1.00	8.06	273	22	75 ± 3	740 ± 20	12.4 ± 0.7	1, 3ii
**2**	4.90/1.40/1.00	8.21	273	22	80 ± 8	760 ± 20	12.0 ± 0.8	2a, 3iii
**3**	4.92/1.40/1.00	8.06	272	23	80 ± 9	750 ± 20	12.9 ± 0.9	2b, 3i
**4**	4.93/1.40/1.00	8.05	272	22	84 ± 10	750 ± 20	13.0 ± 0.9	2c, 3iv
**5**	4.93/1.41/1.00	8.09	272	22	77 ± 9	750 ± 20	12.2 ± 0.7	S14, S15
**6**	4.92/1.41/1.00	8.04	272	21	78 ± 8	740 ± 20	12.7 ± 0.8	4, S16, S22–25
**7**	4.92/1.40/1.00	8.05	271	21	82 ± 8	750 ± 20	13.1 ± 0.6	5, S28, S29
**8**	4.95/1.40/1.00	8.12	273	22	85 ± 9	750 ± 20	12.6 ± 0.8	S35b,c
Non-standard Conditions								
**NS1**	4.93/1.41/1.00	8.13	200	19	81 ± 4	880 ± 20	15 ± 1	S26, S27
**NS2**	4.95/1.41/1.00	4.96	276	35	73 ± 7	770 ± 20	13.0 ± 0.9	S9, S37

[a]Based on CuCl used in the synthesis. Cu-atom content for Syntheses 1 and NS1 was determined following quantitative digestion of the nanocrystals in nitric acid, after which the Cu^{2+}-concentration was determined via the $^2E_g \rightarrow {}^2T_{2g}$ absorption. This analysis provided an extinction coefficient from Synthesis 1 of $\varepsilon_{3.1\mathrm{eV}}^{\mathrm{Cu}} = 2100 \pm 200 \frac{\mathrm{L}}{\mathrm{mol\ Cu} \cdot \mathrm{cm}}$, which was used to determine the copper content and corresponding yield for Syntheses 2–8 and NS2.

[b]As nanoplatelet height can sometimes be difficult to analyze due to orientation bias on the TEM grid, we use the lateral dimension as an indicator for size-reproducibility.

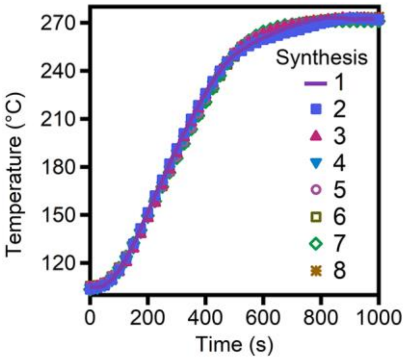

Figure S1. Temperature profiles for the standard $Cu_{3-x}P$ nanocrystal syntheses (Syntheses 1–8). Reactions were heated with a contact voltage regulator. The maximum ramp-rate of dT/d$t \approx 22$ °C/min and asymptotic temperature of $T_f \approx 270$ °C were reproducibly achieved.

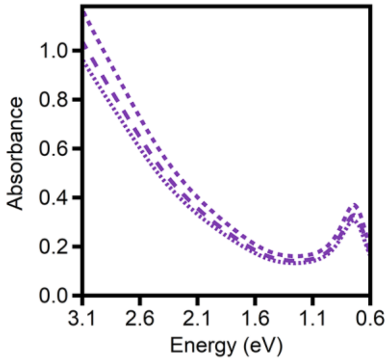

Figure S2. Three independently measured absorption spectra of as-synthesized $Cu_{3-x}P$ nanocrystals for Synthesis 1 (Figure 1). Each spectrum was collected by adding 10 µl stock solution to 790 µl toluene/THF (39/40 by volume).

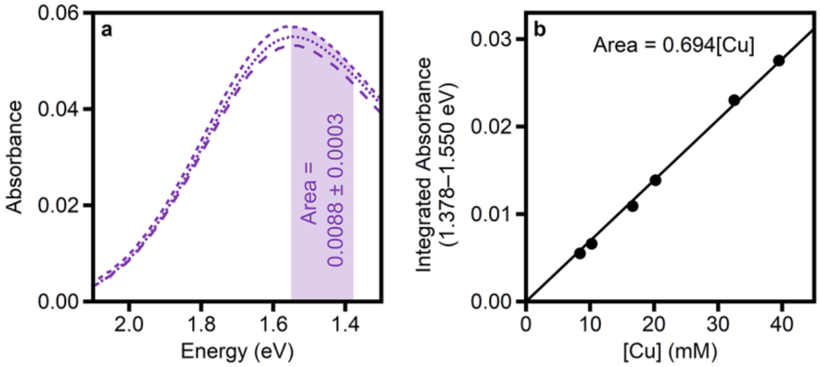

Figure S3. (a) Absorption spectra of three solutions of digested $Cu_{3-x}P$ nanocrystals from Synthesis 1 (Figure 1). Each digestion solution was prepared by the addition of 800 µl nitric acid to a purified $Cu_{3-x}P$ nanocrystal pellet prepared from 60 µl stock solution. The average integrated area (1.378–1.550 eV) is 0.0088 ± 0.0003. **(b)** Calibration line used for the quantification of [Cu^{2+}] in nitric acid. This relationship was used to determine Cu-content in digested nanocrystals.

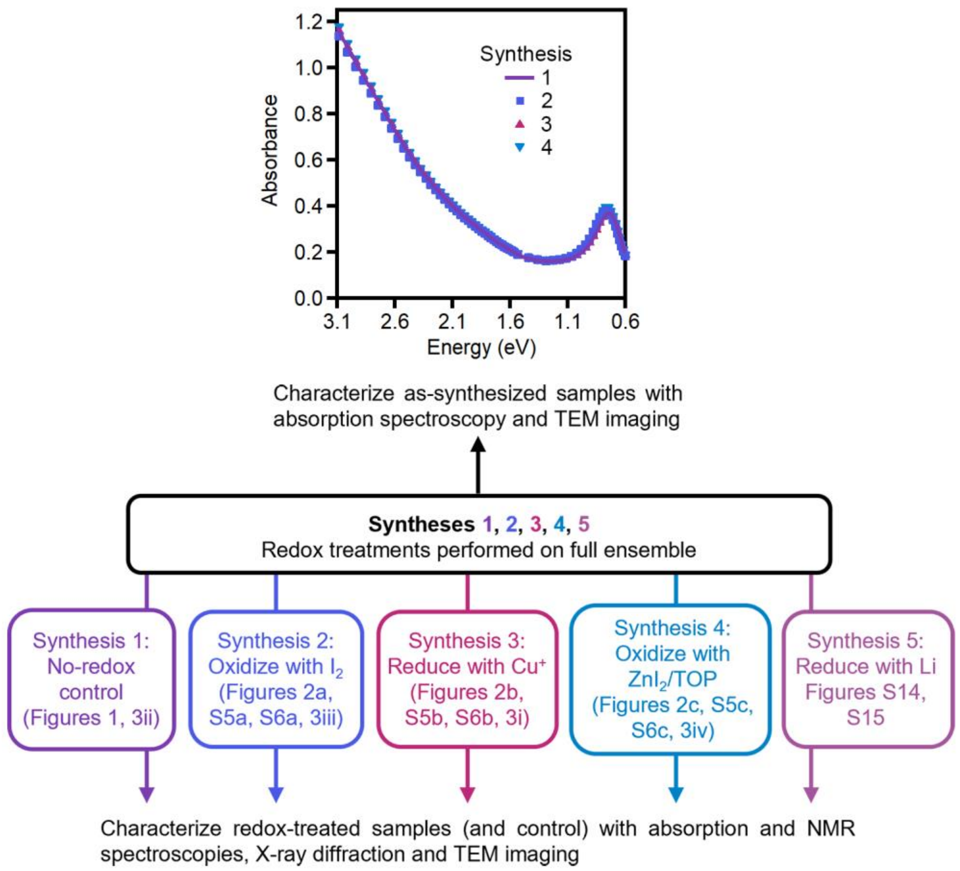

Scheme S1. Workflow for various redox treatments. A reproducible standard synthesis leads to nearly identical average absorption spectra, allowing for inter-ensemble comparisons. Select related Figures are indicated in the Scheme. Syntheses 6–8 and NS1 and NS2 were used for supplemental experiments.

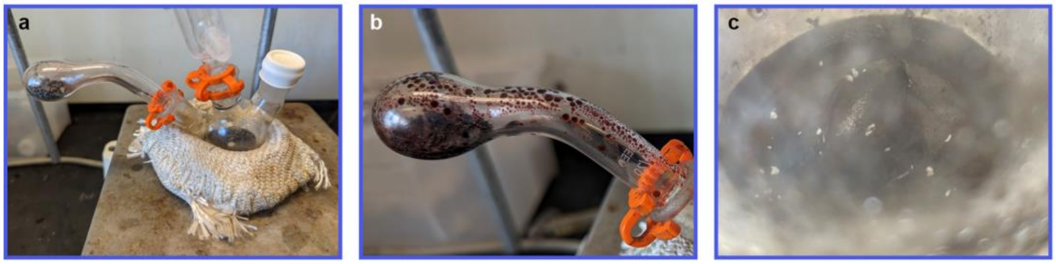

Figure S4. Photographs of the oxidation of $Cu_{3-x}P$ nanocrystals with I_2. **(a)** The setup provides physical separation between the nanocrystal suspension (in toluene) in the round-bottom flask and the iodine solid in the Merlic adapter. **(b)** Red co-condensate droplets of toluene and iodine in the Merlic adapter observed at intermediate times. **(c)** White copper iodide (CuI) solids formed after evacuating the flask at the end of the oxidation.

Table S2. Cu-content and LSPR shifts for $Cu_{3-x}P$ nanocrystal ensembles following various redox treatments.

Synthesis	Redox Treatmen	LSPR Shift (meV)	Post-Redox Cu [illegible]	Change in Cu-Content (%)[b]	Figure
2	I_2	+80 ± 30	93 ± 10	–6.6	2a
3	Cu^+	−50 ± 30	94 ± 13	+2.5	2b
4	ZnI_2/TOP	+140 ± 20	74 ± 12	NA	2c

[a]Based on estimated initial Cu-content from post-synthesis yields of Table S1. Cu-atom content was determined via the $^2E_g \rightarrow ^2T_{2g}$ absorption of solutions made from digested pellets collected after post-redox purification.

[b]Based on analysis of the supernatant. Calculated as % of Cu in as-synthesized nanocrystals; determined via the $^2E_g \rightarrow ^2T_{2g}$ absorption of supernatant.

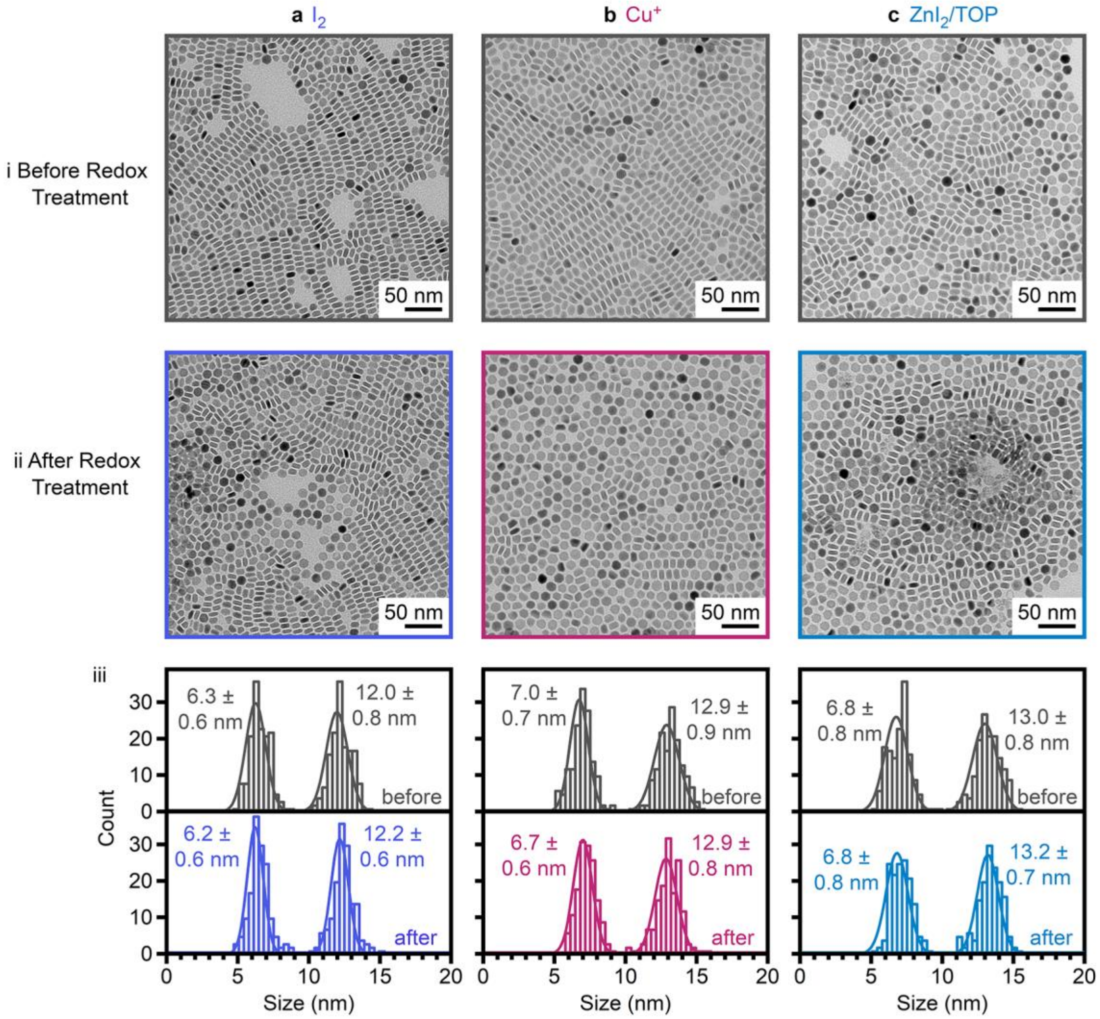

Figure S5. TEM characterization of $Cu_{3-x}P$ nanocrystals (i) before and (ii) after **(a)** oxidation with I_2 **(b)** reduction with Cu^+ and **(c)** oxidation with ZnI_2 and TOP**.** (iii) Statistical analyses (n = 150) of the nanoplatelet lateral dimension and height before (top) and after (bottom) each of the treatments.

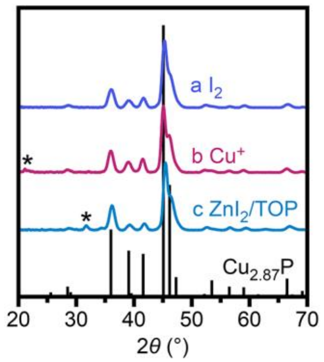

Figure S6. Powder X-ray diffraction patterns of $Cu_{3-x}P$ nanocrystal ensembles after **(a)** oxidation with I_2, **(b)** reduction with Cu^+ and **(c)** oxidation with ZnI_2/TOP. The simulated pattern for $P6_3cm$ $Cu_{2.87}P$ (ref. 1) is shown for comparison. Reflections marked with an asterisk in traces b and c are unindexed reflections observed following treatments containing $[Cu(MeCN)_4]PF_6$ and zinc halides, respectively.

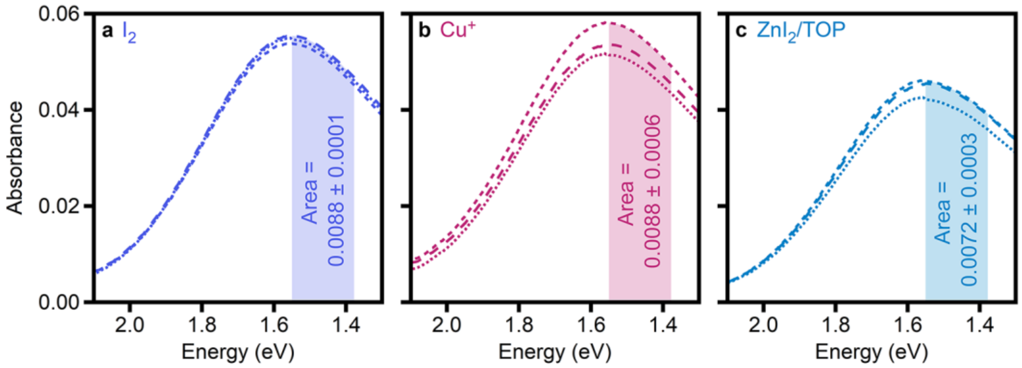

Figure S7. Absorption spectra of Cu^{2+} in nitric acid from digestions of $Cu_{3-x}P$ nanocrystals after **(a)** oxidation with I_2, **(b)** reduction with Cu^+ and **(c)** oxidation with ZnI_2/TOP. These data were used for determination of the post-redox Cu-content (Table S2). For each treatment, 3 pellets were digested and the integrated absorbance (1.378–1.550 eV, shaded region) was used. Each spectrum was collected after addition of 800 µl nitric acid to a purified $Cu_{3-x}P$ nanocrystal pellet prepared from 60 µl stock solution. The small discontinuity sometimes observed near 1.550 eV is due to a change in detectors between the near-IR and visible regions.

Section S1. Analysis of the supernatant following oxidation with I_2

According to previous redox studies[2-4] on copper chalcogenide nanocrystals, CuI forms as an oxidation byproduct when extracted Cu reacts with reduced I_2 (Equation S1):

$$I_2 + 2Cu^+ + 2e^- \rightarrow 2CuI \tag{S1}$$

Following oxidation with I_2 (Figure 2a), the supernatants from all purification steps were combined and concentrated under vacuum to give a waxy white residue (20.2 mg). This residue was digested in nitric acid (2000 µl) to quantify the amount of CuI. The absorption spectrum of the digested supernatant residue (Figure S8a) reveals the characteristic Cu^{2+} peak ($E \approx 1.6$ eV). Quantitative analysis of this peak yields an estimate of 0.043 mmol Cu, corresponding to 6.6 % of the post-synthesis Cu (Table S1). This loss of Cu into the supernatant is in good agreement with the 93 ± 10 % retention of Cu in the nanocrystals based on digestion (Table S2). This calculation suggests that the residue is 41 %-by-mass CuI.

The iodide anion in the supernatant residue was also oxidized by nitric acid. Control absorption measurements were performed to verify that the peak at $E = 2.54$ eV from iodine has no spectral overlap with the 1.378–1.550 eV region used for Cu^{2+}-quantification. The absorption spectra of nitric acid solutions of I_2 and OAm (Figure S8bi), I_2 only (Figure S8bii), and OAm only (Figure S8biii) all indicate negligible absorption from 1.378–1.550 eV. Using an extinction coefficient based on I of $\varepsilon^{\mathrm{I}}_{2.54\mathrm{eV}}$ = 174 L/mol I•cm (derived from Figure S8bi), the supernatant residue is estimated to contain I/Cu = 0.97. This suggests (1) that almost all Cu atoms in the supernatant residue were present as CuI and (2) that the post-oxidation vacuum step removes excess I_2 from the reaction flask.

After a different ensemble (Synthesis NS2) was oxidized with I_2, the supernatant residue was confirmed to contain CuI by powder X-ray diffraction (Figure S9).

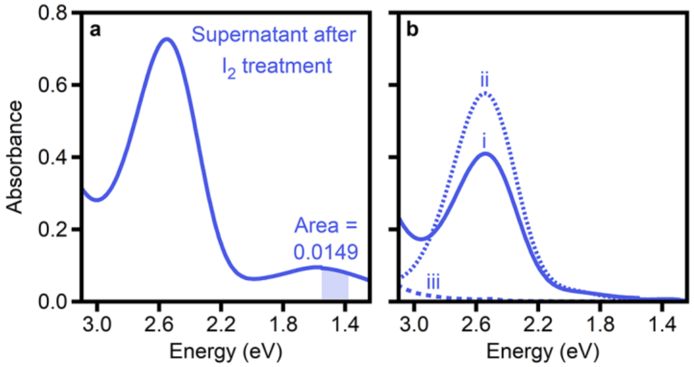

Figure S8. Absorption spectra of **(a)** supernatant residue digested in nitric acid and **(b)** control solutions of (i) 0.0083 M I_2 and 0.042 M OAm, (ii) 0.0083 M I_2, and (iii) 0.042 M OAm.

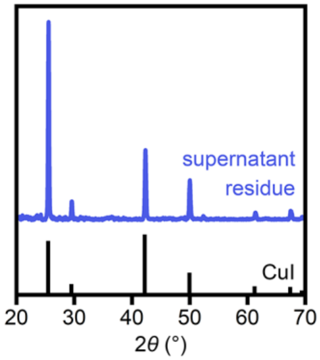

Figure S9. Powder X-ray diffraction pattern of the supernatant residue collected following oxidation of $Cu_{3-x}P$ nanocrystals with I_2 (using the ensemble from Synthesis NS2) compared to that simulated for sphalerite CuI (ref. 5).

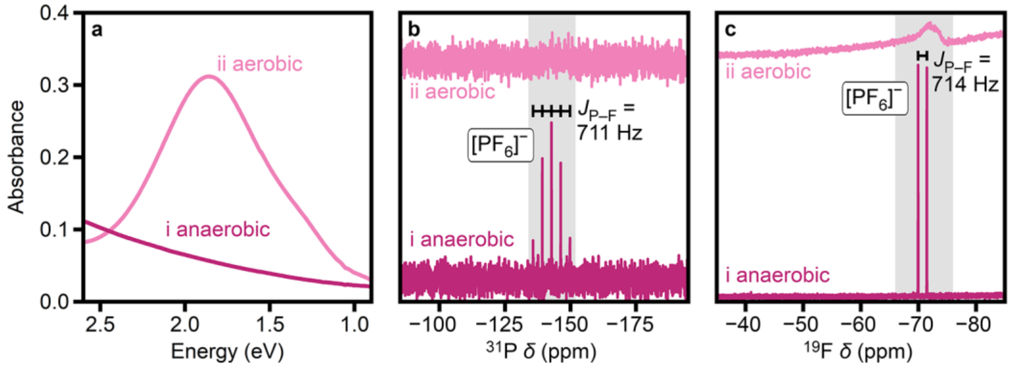

Figure S10. (a) Absorption, **(b)** ^{31}P-NMR and **(c)** ^{19}F-NMR spectra of the supernatant oil from nanocrystal purification following reduction with Cu^{+} (i) before and (ii) after exposure to air. The oil was prepared by concentrating the collected supernatant to 730 µl. For absorption spectroscopy, the solution was prepared by adding 100 µl oil to 500 µl OAm/THF (1/5 by volume). For NMR spectroscopy, the solution was prepared by adding 100 µl oil to 600 µl benzene-d_6. The anaerobic spectra match that expected for free $[PF_6]^-$.[6-8]

Section S2. Analysis of the supernatant following reduction with Cu^+

The supernatant from purification of $Cu_{3-x}P$ nanocrystals after reduction with Cu^+ was concentrated under vacuum to give a colorless oil, consisting of mostly OAm and colorless crystals that formed at ambient temperature. It was anticipated that this oil could be analyzed for Cu^{2+} to quantify the amount of Cu inserted into the $Cu_{3-x}P$ nanocrystals, according to the findings of a previous study in which $Cu_{1.1}S$ nanocrystals are said to be reduced at the expense of some oxidation of Cu^+ to Cu^{2+}.[9] The expected balanced redox reactions for the analogous mechanism are:

$$Cu_{3-x}P + \gamma Cu^+ + \gamma e^- \rightarrow Cu_{3-x+\gamma}P \tag{S2}$$

$$\gamma Cu^+ \rightarrow \gamma Cu^{2+} + \gamma e^- \tag{S3}$$

$$Cu_{3-x}P + 2\gamma Cu^+ \rightarrow Cu_{3-x+\gamma}P + \gamma Cu^{2+} \tag{S4}$$

Surprisingly, the anaerobic supernatant oil revealed no Cu^{2+} peak by absorption spectroscopy (Figure S10i). This observation does not rule out the previously established reduction mechanism, as the byproduct Cu^{2+} species may have been subsequently reduced. It is possible that Cu^{2+} generated from the reduction was subsequently reduced by the majority solvent THF, based on its precedence as an electron donor.[10] Regardless, we thus cannot simply quantify Cu^{2+} to deduce inserted Cu.

When air was bubbled through the solution containing the supernatant oil, it turned clear blue. To enable quantitative analysis of this absorption spectrum, a control solution ([Cu] = 0.022 M) was prepared by adding $[Cu(MeCN)_4]PF_6$ (36 mg, 0.098 mmol) to OAm (730 µl) and heating at 100 °C under vacuum to remove MeCN. From this mixture, 100 µl was added to 500 µl THF. The colorless solution turned clear blue immediately with air-bubbling. The absorption spectrum (Figure S11a) was used to determine an extinction coefficient of $\varepsilon^{Cu}_{1.88eV}$ = 82 L/mol Cu•cm. Analysis of the supernatant absorption spectrum (Figure S10ii) using this extinction coefficient indicates only 0.083 mmol Cu remained in the supernatant, which would correspond to a loss of 45 % of the Cu from $[Cu(MeCN)_4]PF_6$. Considering the limited solubility of $[Cu(MeCN)_4]PF_6$, however, it is unreasonable to assume that the full amount of missing Cu was inserted into $Cu_{3-x}P$ nanocrystal lattice.

To estimate the amount of Cu lost from the supernatant (and thus inserted into the $Cu_{3-x}P$ nanocrystals), a control experiment was used to determine the extent of co-precipitation of molecular Cu. The procedure for reduction with Cu^+ was followed with the exclusion of $Cu_{3-x}P$ nanocrystals. Centrifugation was used to collect a beige solid instead of the usual $Cu_{3-x}P$ nanocrystal pellet. The absorption spectrum from this control (Figure S11b) was measured identically to the supernatant from the reduction experiment and revealed that only 65 % of the initial Cu remained in solution. Using the assumption that the same 35 % proportion of molecular Cu co-precipitated with $Cu_{3-x}P$ nanocrystals, it is estimated that 0.015 mmol Cu was inserted into $Cu_{3-x}P$ nanocrystals, corresponding to 2.5 % increase in Cu-content.

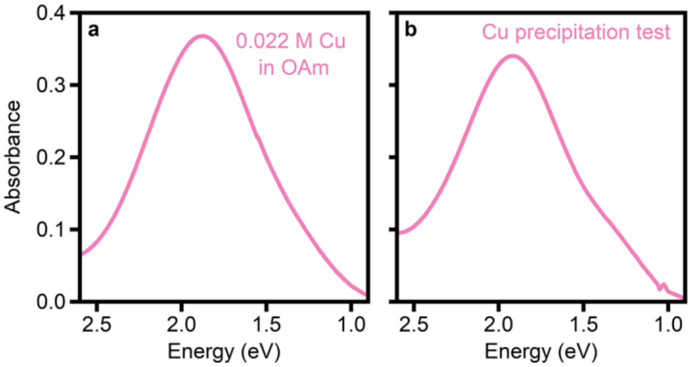

Figure S11. Aerobic absorption spectra of **(a)** control solution with [Cu] = 0.022 M and **(b)** supernatant oil from co-precipitation test. Both spectra were collected in OAm/THF (1/5 by volume).

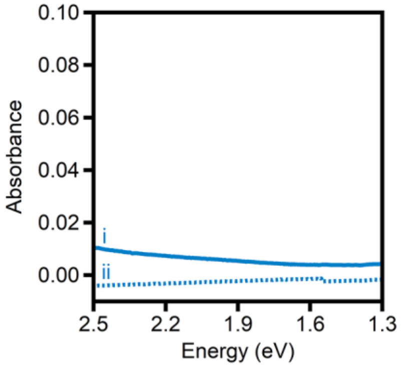

Figure S12. Aerobic absorption spectra of: (i) the concentrated supernatant oil following oxidation with ZnI_2/TOP and (ii) a control solution with CuI and TOP ([Cu] = 0.0063 M, TOP/Cu = 52). Both spectra were collected using TOP/THF (1/5 by volume) as the solvent.

Section S3. Analysis of the supernatant following oxidation with ZnI_2 and TOP

Unlike the redox experiments using I_2 and Cu^+, the ZnI_2/TOP experiment has no intuitive or literature basis for being a redox reaction. The substantial LSPR blueshift (Figure 2c), lattice contraction (Figure 3aiv), and downfield ^{31}P-NMR Knight shift (Figure 3b), however, collectively suggest that the ZnI_2/TOP treatment yields a Cu-coupled oxidation of the nanocrystals. Such an oxidation would be expected to lead to an extraction of Cu atoms from nanocrystals into the supernatant.

The supernatant collected during purification after treatment with ZnI_2 and TOP was colorless, as expected for a solution with the d^{10} metal ions Cu^+ and Zn^{2+}. Efforts to oxidize the evaporated supernatant oil in air (as done in Section S2) were unsuccessful (Figure S12i), likely due to the propensity of Cu complexes with alkylphosphine ligands to adopt a Cu^+ oxidation state. Attempts to oxidize the oil with nitric acid (as done in Section S1) failed due to the immiscibility between TOP and nitric acid. These limitations precluded an optical analysis of supernatant Cu content by Cu^{2+} quantification.

An alternative analysis was devised to support the assertion of Cu atom extraction from the oxidized $Cu_{3-x}P$ nanocrystals (for the experiment corresponding to Figure 5i), using solution ^{31}P-NMR spectroscopy to empirically determine how Cu perturbs the TOP-derived resonance in the presence of excess Zn/Cu. The supernatant oil was diluted with benzene-d_6 in a 1:6 volume ratio. Four controls were prepared with 4.0 TOP/ZnI_2, three with varying [Zn] and no Cu and the fourth containing 0.083 CuI/ZnI_2 ([Cu + Zn] = 6.8 mM). A sharper peak at −30.4 ppm from unbound TOP[11] is present in the ^{31}P-NMR spectra (Figure S13) of the four control solutions and the supernatant, as expected. An additional broader peak, likely from bound TOP, is also present in all three spectra but with a chemical shift which appears to vary with solution Cu content. The most upfield shift of −31.5 ppm was observed with the Cu-free standards (Figure S13i–iii) and the most downfield shift of −30.9 ppm with the 0.083 Cu/Zn ratio standard (Figure S13iv). If this shift of the broad peak arises from the presence of Cu, the intermediate chemical shift of −31.2 ppm for the supernatant broad peak (Figure S13v) implies that the supernatant has Cu/Zn < 0.083.

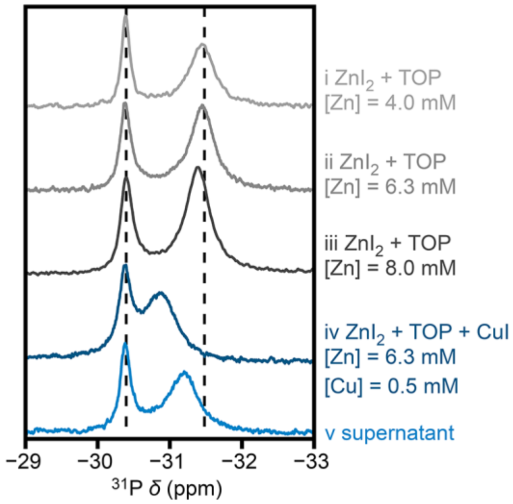

Figure S13. ^{31}P-NMR spectra of Cu-free control solutions with TOP/Zn = 4.0 and (i) [Zn] = 4.0 mM, (ii) [Zn] = 6.3 mM and (iii) [Zn] = 8.0 mM. (iv) Supernatant following oxidation with ZnI_2/TOP ([TOP] ≈ 24 mM). (v) Control solution with [Zn] = 6.3 mM and [Cu] = 0.5 mM (TOP/Zn/Cu = 4.0/1.0/0.083). The dashed lines are guides to the eye.

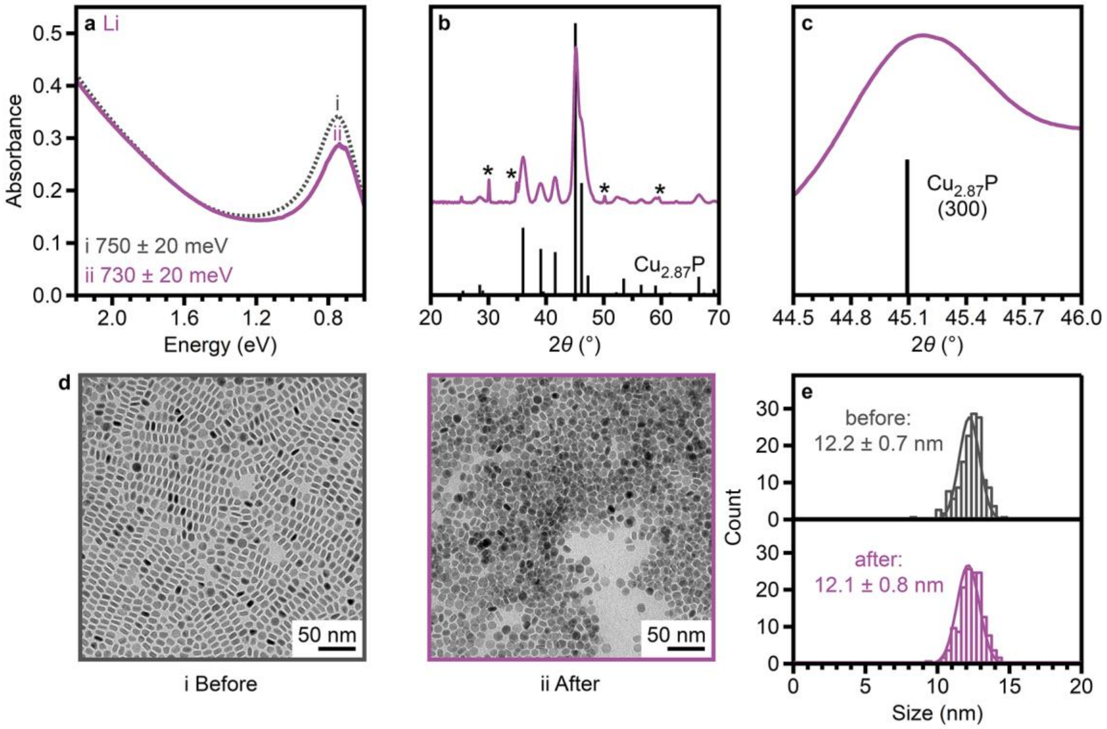

Figure S14. (a) Average absorption spectra of $Cu_{3-x}P$ nanocrystals (i) before and (ii) after reaction with Li in toluene/THF (9/1 by volume). The loss of intensity without a redshift of the LSPR is indicative of slight aggregation without reduction. **(b)** Comparison of the powder X-ray diffraction pattern of the Li-treated nanocrystals with the simulated pattern for $P6_3cm$ $Cu_{2.87}P$ (ref. 1) confirms that the phase is maintained. Asterisks indicate LiCl reflections.[12] **(c)** The lack of shift in the (300) reflection confirms that the nanocrystals are not reduced. **(d)** TEM images of $Cu_{3-x}P$ nanocrystals (i) before and (ii) after reaction with Li in toluene/THF (9/1 by volume). **(e)** Statistical analysis of the lateral dimension from the TEM images. Orientation bias precluded statistical analysis of the nanoplatelet height.

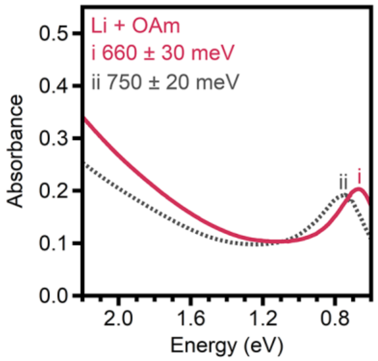

Figure S15. Absorption spectra of $Cu_{3-x}P$ nanocrystals after reduction with Li in the presence of 1.5 OAm/Cu after (i) 30 min and (ii) 48 h of storage in Li-free toluene. The spectrum for as-synthesized nanocrystals (Synthesis 5) is presented in Figure S14ai.

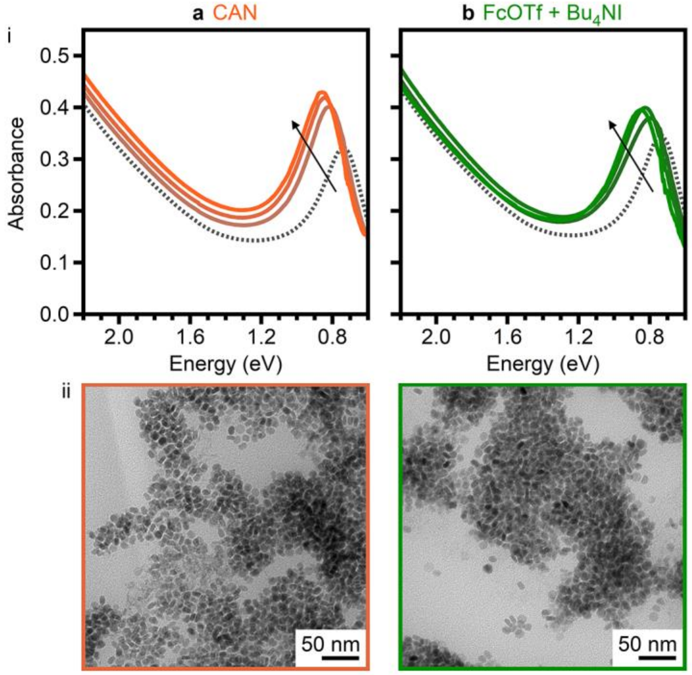

Figure S16. (i) Absorption spectra and (ii) TEM images following treatment of $Cu_{3-x}P$ nanocrystals with **(a)** $(NH4)_2Ce(NO_3)_6$ (CAN; up to 0.43 CAN/Cu) or **(b)** ferrocenium triflate and tetrabutylammonium iodide ($FcOTf + Bu_4NI$; 0.25/0.28/1 $FcOTf/Bu_4NI/Cu$). Arrows show the direction of increasing amounts of oxidant.

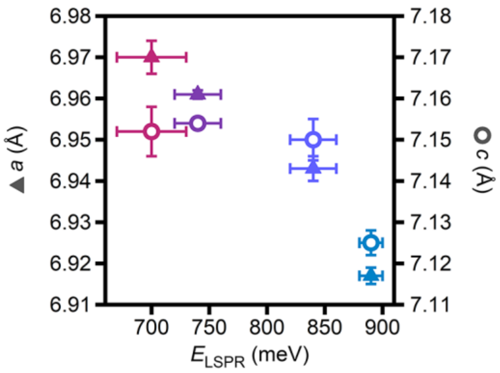

Figure S17. Unit-cell parameters, *a* (left, triangles), and *c* (right, circles), as a function of LSPR energy.

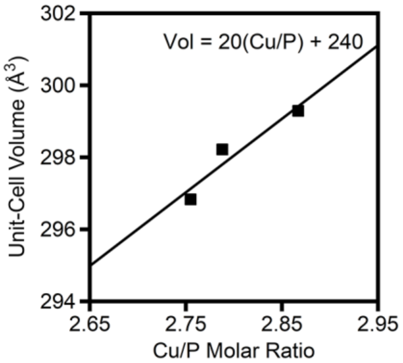

Figure S18. Unit-cell volume as a function of composition for bulk $Cu_{3-x}P$ (ref. 1). The solid line is a best fist to the data, which was used to calculate the Cu/P molar ratios reported in Tables 1 and S5.

Section S4. Drude–Lorentz modeling of optical data

In nanocrystals, the observed LSPR frequency, ω_{sp}, correlates with charge carrier concentration, N_h, the dielectric constant, ε_m, of the solvent environment and the carrier collision frequency, γ (Equation S5). With the assumption that the dielectric function of $Cu_{3-x}P$ in the LSPR region is dominated by free carriers,[13] the bulk plasma frequency, ω_p, can be calculated using Equation S5:

$$\omega_p = \sqrt{\left(\omega_{sp}^2 + \gamma^2\right)(1 + 2\varepsilon_m)} \quad \text{(S5)}$$

For these calculations, γ is experimentally estimated as the full width at half maximum of the LSPR absorption. For the toluene/THF (1/1 by volume) solvent system used for optical measurements in this study, ε_m is approximated as 5.33, which is the corresponding arithmetic molar average of the dielectric constants for toluene (2.38) and THF (7.58). Using the as-synthesized example from Figure 1d (ω_{sp} = 744 meV, γ = 233 meV), Equation S5 yields ω_p = 2.66 eV. N_h can be calculated from ω_p and m_h* using Equation S6:

$$N_h = \left(\omega_p^2 \varepsilon_0 m_h^*\right) / e^2 \quad \text{(S6)}$$

The effective hole mass, m_h* is approximated as 0.67m_e, where m_e is the electron mass, which was determined by taking the harmonic mean of the calculated *ab*-plane and *c*-axis effective masses.[14] Continuing with the use of the as-synthesized example, Equation S6 yields an estimate of $N_h = 3.4 \times 10^{21}$ cm^{-3}. Drude–Lorentz calculations using the high frequency dielectric constant, ε_∞,[15] which is approximated as 2.5 from DFT-calculated dielectric function at photon energy = 7 eV for bulk $Cu_{3-x}P$,[14] yield an estimate of $N_h = 3.9 \times 10^{21}$ cm^{-3}. The 13 % increase is sufficiently small that Equation S5 is used for calculation of bulk plasma frequency and delocalized hole concentrations reported in Table 1.

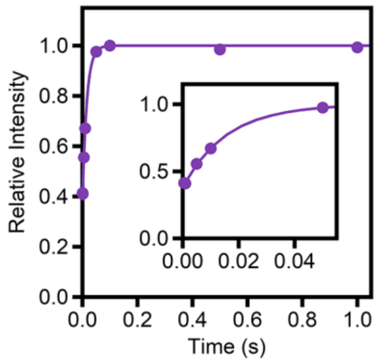

Figure S19. Saturation-recovery experiments of as-synthesized $Cu_{3-x}P$ nanocrystals performed at 186 ppm and T = 300 K. The solid line is a fit to the equation $y = 1 - e^{-t/T_1}$.

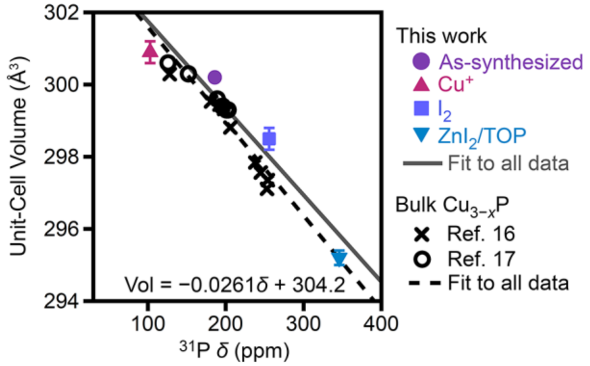

Figure S20. Unit-cell volume as a function of solid-state ^{31}P MAS NMR chemical shift. The solid gray line is a fit to all collected data. Additional data as a function of chemical shift and corresponding fits are presented for bulk $Cu_{3-x}P$ from refs. [16] and [17].

Table S3. Relationship between unit-cell volume and ^{31}P chemical shift.

Redox Treatment	Unit-Cell Volume (Å^3)	Expected Isotropic δ (ppm)[a]	Most Intense Resonance (ppm)
As-synthesized	300.2	153.3	186
Cu	300.9	126.4	103
I_2	298.5	218.4	256
ZnI_2/TOP	295.2	344.8	346

[a]Based on the empirically derived relationship Vol = $-0.0261\delta + 304.2$ (Figure S20).

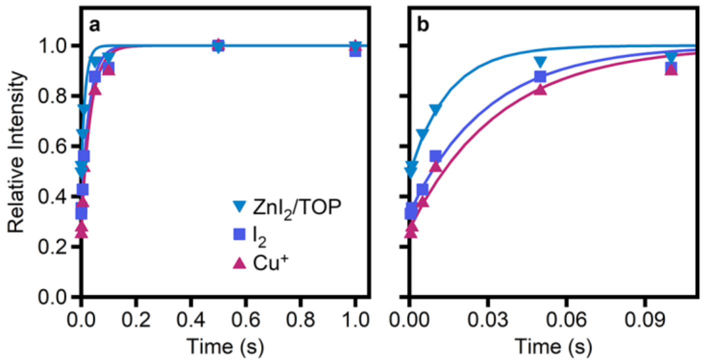

Figure S21. ^{31}P-NMR saturation–recovery experiments at T = 300 K for redox-treated $Cu_{3-x}P$ nanocrystals. Data are collected at the fields indicated by arrows in Figure 3c. The solid lines are fits to the equation $y = 1 - e^{-t/T_1}$. Data/fits are shown **(a)** at full-scale and **(b)** magnified at short times.

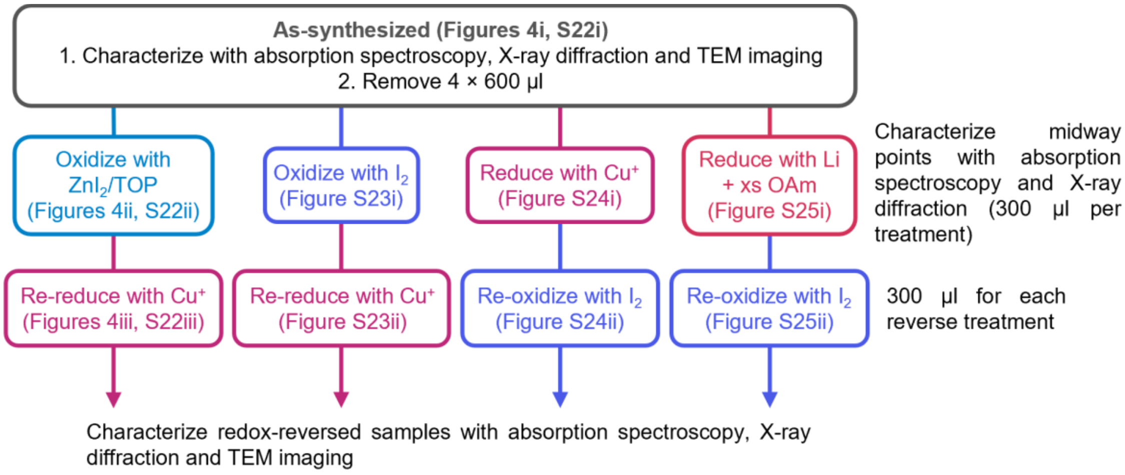

Scheme S2. Workflow of redox reversibility experiments. These experiments were performed on the ensemble from Synthesis 6.

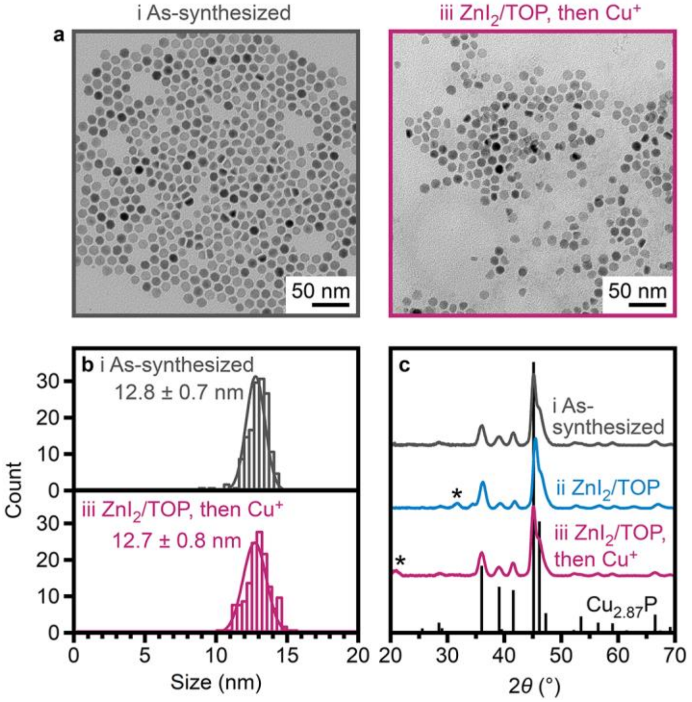

Figure S22. **(a)** TEM images, **(b)** corresponding statistical analysis of the lateral dimension and **(c)** X-ray diffraction of $Cu_{3-x}P$ nanocrystals (i) as-synthesized, (ii) oxidized with ZnI_2/TOP and (iii) re-reduced with Cu^+. Orientation bias precluded statistical analysis of the nanoplatelet height. Reflections marked with an asterisk in traces cii and ciii are unindexed reflections observed following treatments containing zinc halides and $[Cu(MeCN)_4]PF_6$, respectively.

Table S4. Collected compositional, optical and structural data for all samples characterized with powder X-ray diffraction.

Primary Figure	Synthesis	Redox Treatment	LSPR Max (meV)	Unit-Cell Volume (Å^3)[a]	Cu/P[a]	ΔCu (%, X-ray)[b]
1	1	As-synthesized	740 ± 20	300.2	2.90	
2a	2	I_2	840 ± 20	298.5	2.82	−2
2b	3	Cu(I)	700 ± 30	300.9	2.93	+1
2c	4	ZnI_2/TOP	890 ± 10	295.2	2.67	−8
S14	5	Li	730 ± 20	300.0	2.89	< 1
4	6	As-synthesized	740 ± 20	300.6	2.91	
		ZnI_2/TOP	860 ± 10	295.6	2.69	−8
		ZnI_2/TOP then Cu(I)	700 ± 30	300.9	2.93	< 1
S23	6	I_2	880 ± 20	299.8	2.88	−1
		I_2 then Cu(I)	770 ± 20	300.7	2.92	< 1
S24		Cu(I)	700 ± 30	301.5	2.95	+1
S25		Li/OAm	660 ± 30	299.3	2.86	−2
S26, S27	NS1	As-synthesized	880 ± 20	298.0	2.80	
		Cu(I)	760 ± 20	300.0	2.89	+3
		ZnI_2/TOP	880 ± 20	296.3	2.72	−3
5	7	As-synthesized	750 ± 20	300.9	2.93	
		ZnI_2/TOP (3.9 Zn/Cu)	850 ± 10	296.6	2.74	−7
		$ZnCl_2$/TOP	840 ± 20	297.4	2.77	−5
		CdI_2/TOP	830 ± 20	297.5	2.77	−5
		$CdCl_2$/TOP	840 ± 20	297.6	2.78	−5
		TOP no MX_2	770 ± 20	299.3	2.86	−3
		ZnI_2 no TOP	750 ± 20	299.5	2.87	−2
		ZnI_2/TOP (0.5 Zn/Cu)	800 ± 20	298.9	2.84	−2
		OAm only	750 ± 20	300.6	2.92	<1

[a]Calculated analogously to that of Table 1 using diffraction data.
[b]Estimated using the relationship between unit-cell volume and Cu/P for bulk $Cu_{3-x}P$ (Figure S18) assuming constant P.

Table S5. Lateral dimension and nanoplatelet heights determined by Scherrer analysis for nanocrystals that were (reversibly) oxidized and re-reduced.

Redox Treatment	Scherrer Lateral Size (nm)	Scherrer Height (nm)
As-synthesized	11.6	5.8
ZnI_2/TOP then Cu(I)	11.8	5.7
I_2 then Cu(I)	12.0	5.7

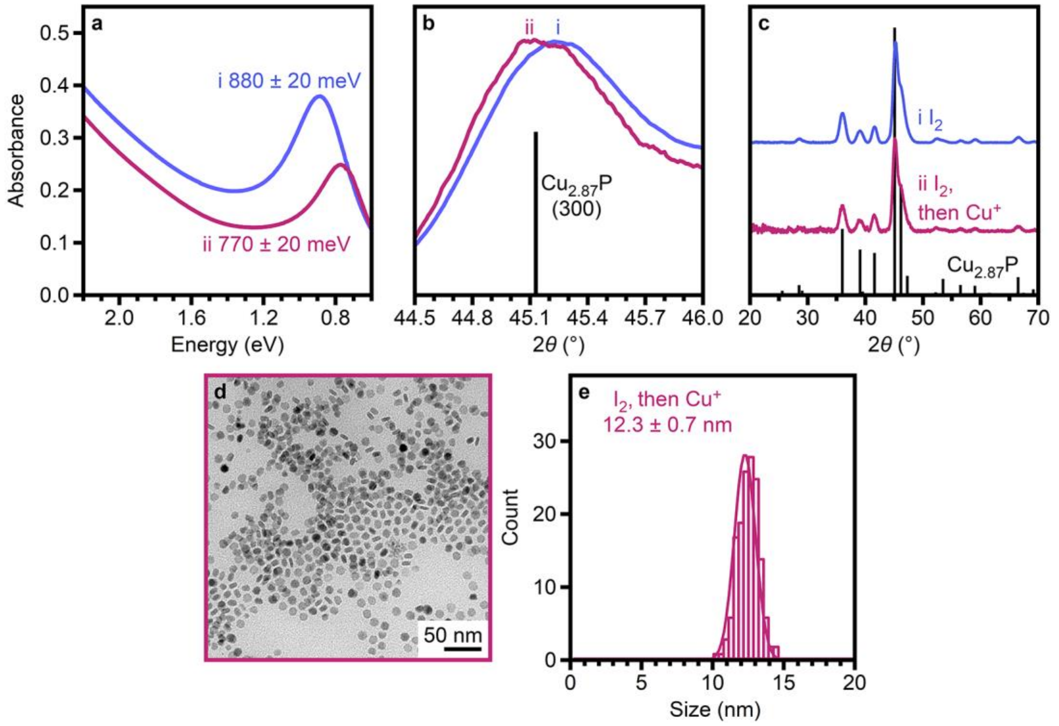

Figure S23. (a) Absorption spectra and **(b)** magnified and **(c)** full-scale powder X-ray diffraction patterns (compared to that simulated for $Cu_{2.87}P$ $P6_3cm$, ref. 1) of $Cu_{3-x}P$ nanocrystals (i) oxidized with I_2 and (ii) re-reduced with Cu^+. **(d)** TEM and **(e)** statistical analysis (n = 150) of the lateral dimension of re-reduced nanocrystals. Orientation bias precluded statistical analysis of the nanoplatelet height. Data for as-synthesized nanocrystals (Synthesis 6) are presented in Figures 4i, S22i.

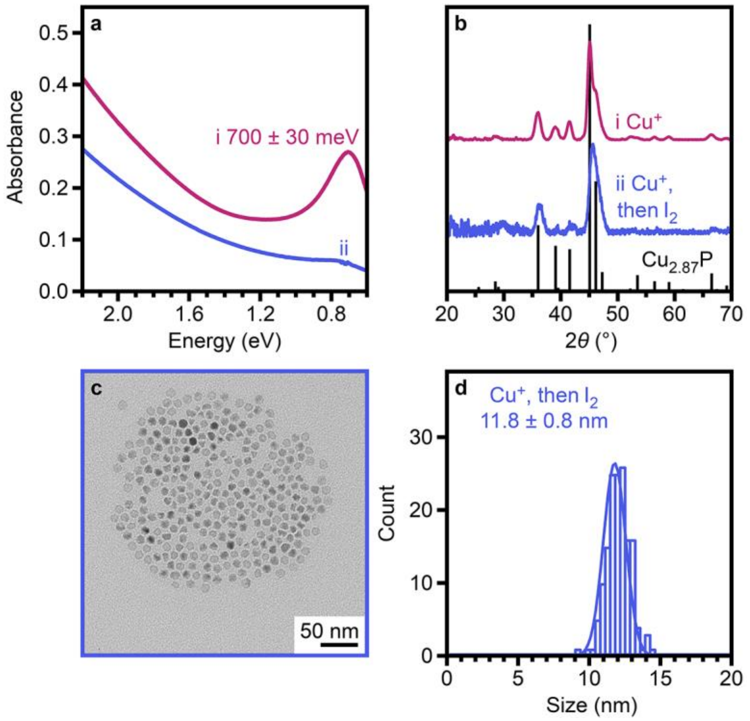

Figure S24. (a) Absorption spectra and **(b)** magnified powder X-ray diffraction patterns (compared to that simulated for $Cu_{2.87}P$ $P6_3cm$, ref. 1) of $Cu_{3-x}P$ nanocrystals (i) reduced with Cu^+ and (ii) re-oxidized with I_2. **(c)** TEM and **(d)** statistical analysis (n = 150) of the lateral dimension of re-reduced nanocrystals. Orientation bias precluded statistical analysis of the nanoplatelet height. Data for as-synthesized nanocrystals (Synthesis 6) are presented in Figures 4i, S22i.

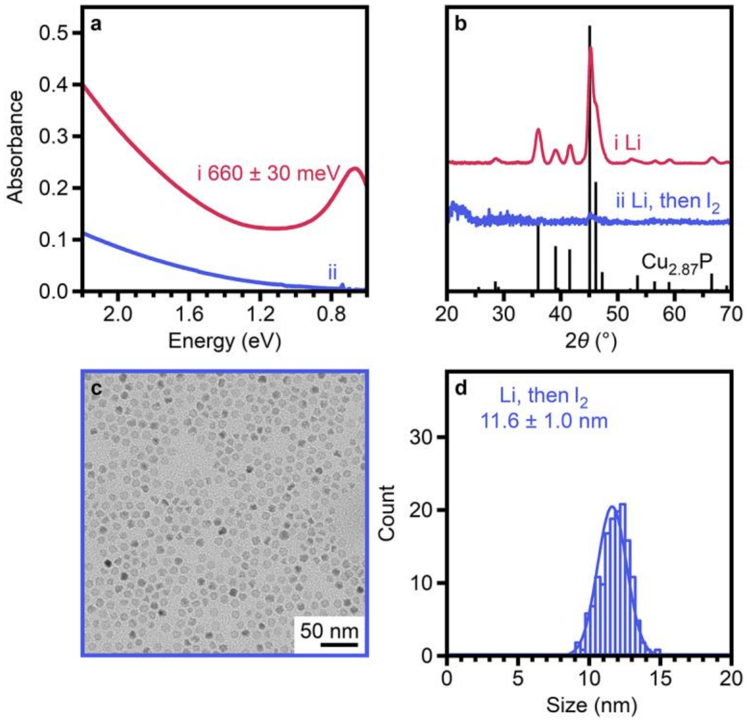

Figure S25. **(a)** Absorption spectra and **(b)** magnified powder X-ray diffraction patterns (compared to that simulated for $Cu_{2.87}P$ P6₃*cm*, ref. 1) of $Cu_{3-x}P$ nanocrystals (i) reduced with Li/OAm and (ii) re-oxidized with I_2. **(c)** TEM and **(d)** statistical analysis (n = 150) of the lateral dimension of re-reduced nanocrystals. Orientation bias precluded statistical analysis of the nanoplatelet height. Data for as-synthesized nanocrystals (Synthesis 6) are presented in Figures 4i, S22i.

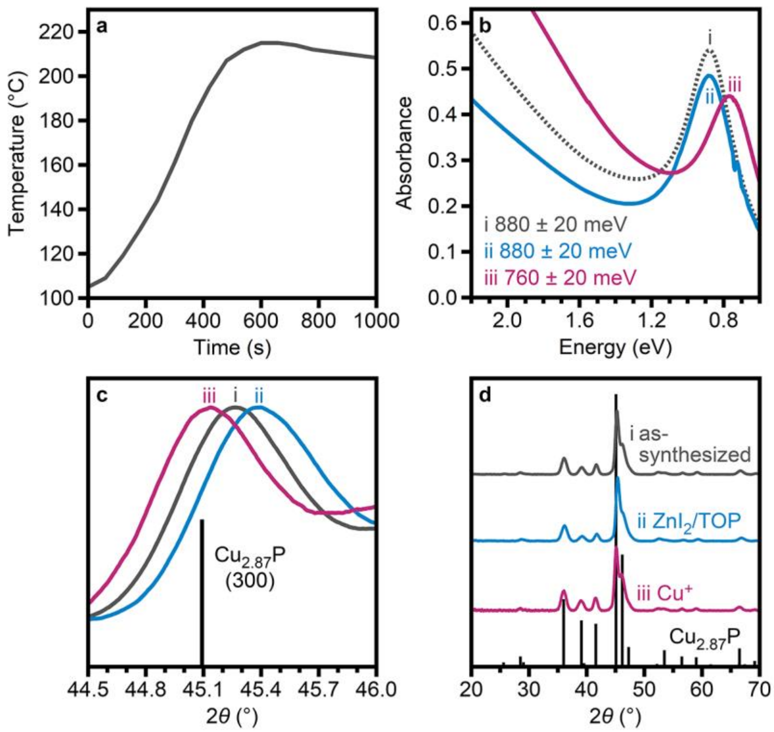

Figure S26. (a) Temperature profile used to synthesize Cu-deficient $Cu_{3-x}P$ nanocrystals**. (b)** Average absorption spectra and **(c)** full-scale and **(d)** magnified powder X-ray diffraction patterns (compared to that simulated from single-crystal data, ref. 1) for Cu-deficient $Cu_{3-x}P$ nanocrystals (i) as-synthesized, (ii) treated with ZnI_2/TOP and (iii) reduced with Cu^+.

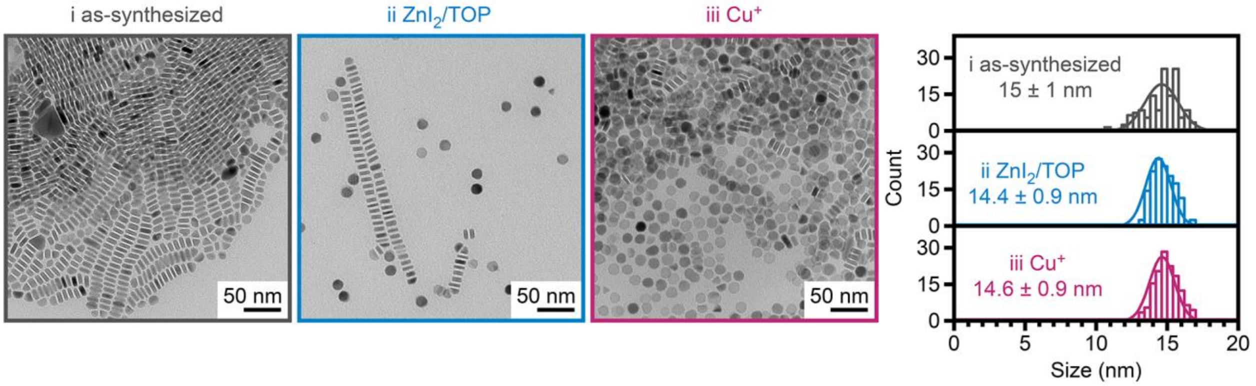

Figure S27. TEM images and statistical analysis of the lateral dimension for Cu-deficient $Cu_{3-x}P$ nanocrystals (i) as-synthesized, (ii) treated with ZnI_2/TOP and (iii) reduced with Cu^+. Orientation bias precluded statistical analysis of the nanoplatelet height.

Table S6. Details and experimental observables of post-synthetic MX_2/TOP ($M^{2+} = Zn^{2+}, Cd^{2+}$; $X^- = Cl^-, I^-, OAc^-$) experiments (Figures 5, S29, S30). All experiments in this table used the ensemble from Synthesis 7. All experiments were performed on nanocrystal suspensions with [Cu] = 0.81 M.

Post-Synthetic Treatment	M/Cu (M = Zn, Cd)	TOP/Cu	OAm/Cu	LSPR Max (eV)	(300) (°)	Scherrer Lateral Size (nm)	Scherrer Height (nm)
None	-	-	-	750 ± 20	45.09 ± 0.01	11.4	5.8
ZnI_2/TOP	3.9	15.5	3.0	850 ± 10	45.35 ± 0.01	11.3	5.7
$ZnCl_2$/TOP	3.8	15.5	3.1	840 ± 20	45.33 ± 0.01	11.1	5.8
CdI_2/TOP	3.8	15.5	2.9	830 ± 20	45.30 ± 0.01	11.3	5.8
$CdCl_2$/TOP	3.8	15.5	3.0	840 ± 20	45.29 ± 0.01	11.1	5.7
1/8 ZnI_2/TOP	0.5	1.9	2.9	800 ± 20	45.22 ± 0.01	11.5	5.8
TOP only (no MX_2)	0	15.5	2.9	770 ± 20	45.19 ± 0.01	11.4	5.8
ZnI_2 only (no TOP)	3.9	0	2.9	750 ± 20	45.15 ± 0.01	11.4	5.8
OAm only	0	0	3.0	750 ± 20	45.11 ± 0.01	11.9	6.3
$Zn(OAc)_2$/TOP	3.8	15.5	2.9	NA	45.21 ± 0.01	10.5	5.6

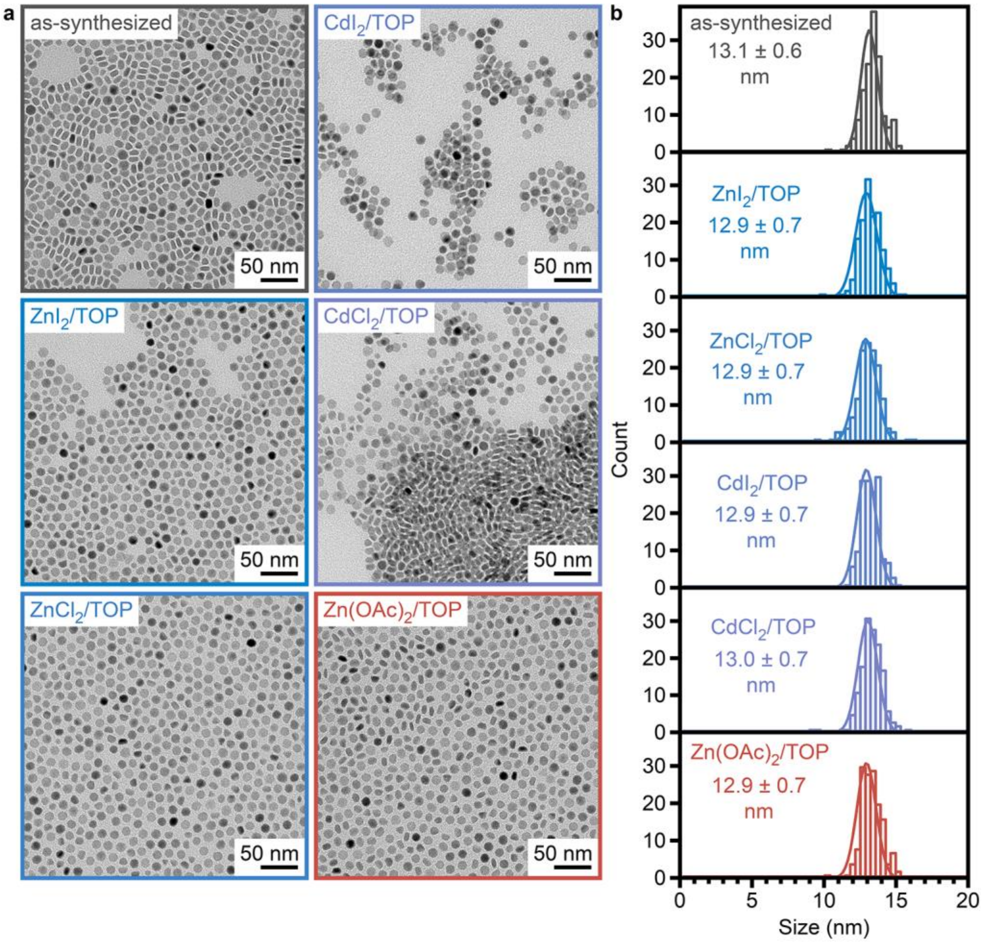

Figure S28. TEM characterization of $Cu_{3-x}P$ nanocrystals treated with various forms of MX_2/TOP ($M^{2+} = Zn^{2+}$, Cd^{2+}; $X^- = Cl^-$, I^-, OAc^-). **(a)** TEM images and **(b)** statistical analyses (n = 150) of the lateral dimension. Orientation bias of some samples precluded statistical analysis of the nanoplatelet height. All experiments from this figure used the ensemble from Synthesis 7.

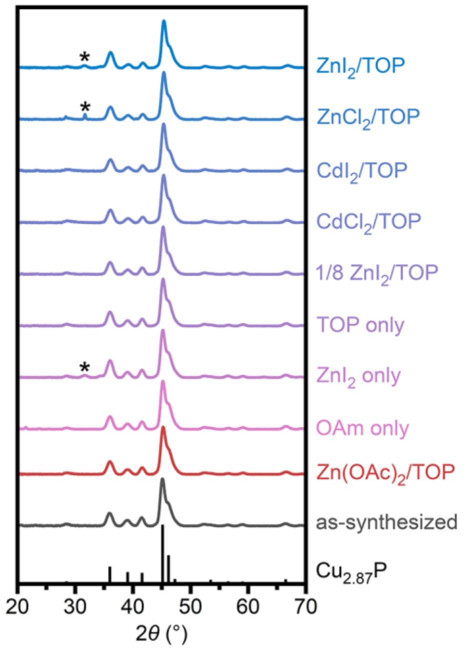

Figure S29. Powder X-ray diffraction patterns of $Cu_{3-x}P$ nanocrystals following various MX_2/TOP treatments. Experimental details are provided in Table S6. The simulated pattern for $P6_3cm$ $Cu_{2.87}P$ (ref. 1) is shown for comparison. Asterisks indicate an unindexed peak observed following treatments containing zinc halides.

Table S7. ICP–MS elemental analysis of select $Cu_{3-x}P$ nanocrystals before and after oxidation with MX_2/TOP.

Redox Treatment	Synthesis	As-Synthesized Cu/P	Post-Redox Cu/P	% Cu Lost[a]	M/P	ΔCu/P	∣M/ΔCu∣
ZnI_2/TOP	6	1.78 ± 0.02	1.51 ± 0.02	15 ± 2	0.20 ± 0.01	−0.27 ± 0.03	0.7 ± 0.1
	7	1.81 ± 0.02	1.60 ± 0.02	12 ± 3	0.20 ± 0.02	−0.22 ± 0.03	0.9 ± 0.2
CdI_2/TOP	7	1.81 ± 0.02	1.51 ± 0.03	17 ± 3	0.14 ± 0.01	−0.30 ± 0.04	0.5 ± 0.1

[a]Calculated with the assumption that P-atom content is unchanged after redox. This assumption is reasonable because the decrease in Cu-atom content by ICP–MS for the Synthesis 7 samples for ZnI_2/TOP and CdI_2/TOP treatments (compared to as-synthesized) are −12 ± 2 % and −14 ± 2 %, respectively.

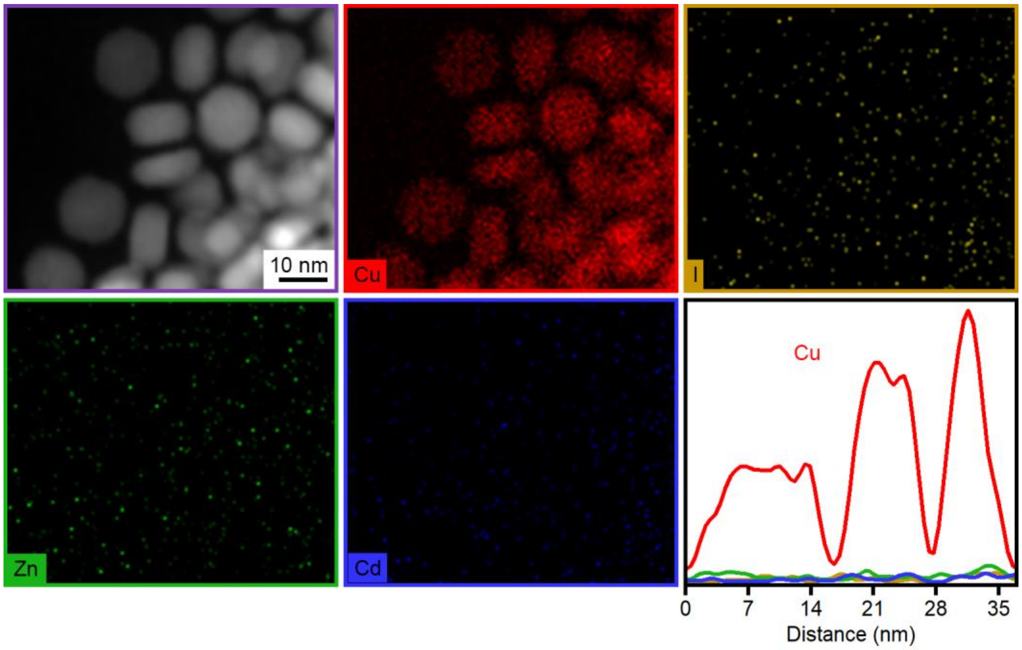

Figure S30. STEM–EDS mapping images and corresponding line-scan of as-synthesized) $Cu_{3-x}P$ nanocrystals (Figure 5ix). Line-scan noise was reduced through binomial smoothing over a 1-nm range of pixels. As expected, the Cu-Kα signal co-localizes with the nanoplatelets from the dark-field STEM image. The background Zn-Kα, Cd-Lα and I-Lα signals are also shown.

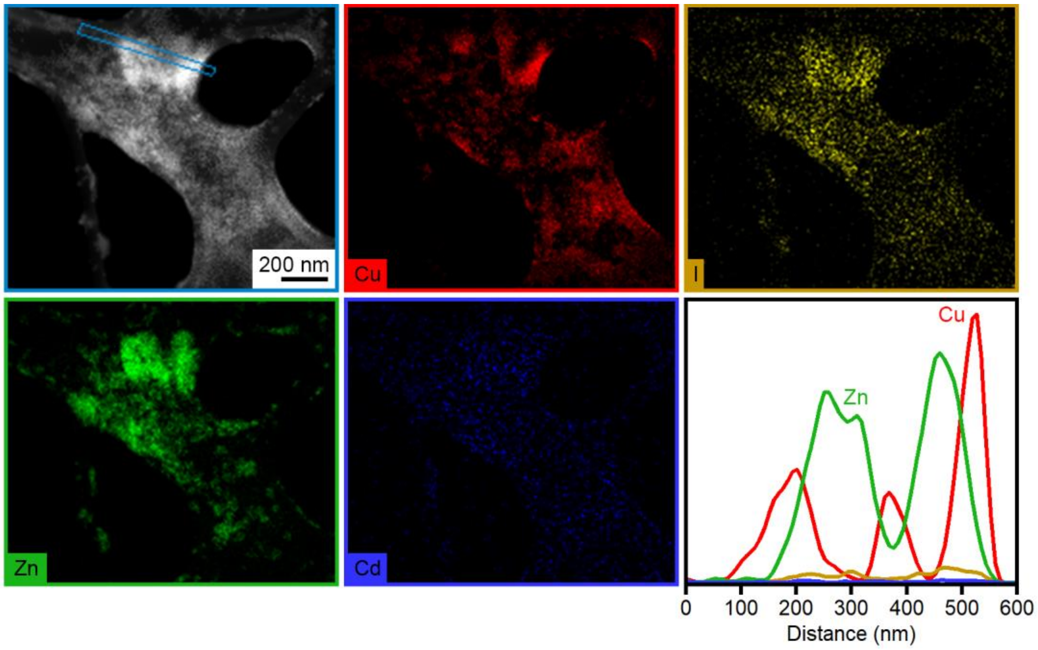

Figure S31. STEM–EDS mapping images and corresponding line-scan of $Cu_{3-x}P$ nanocrystals oxidized with ZnI_2/TOP (Figure 5i). Line-scan noise was reduced through binomial smoothing over a 16-nm range of pixels. Mapping reveals Zn aggregates.

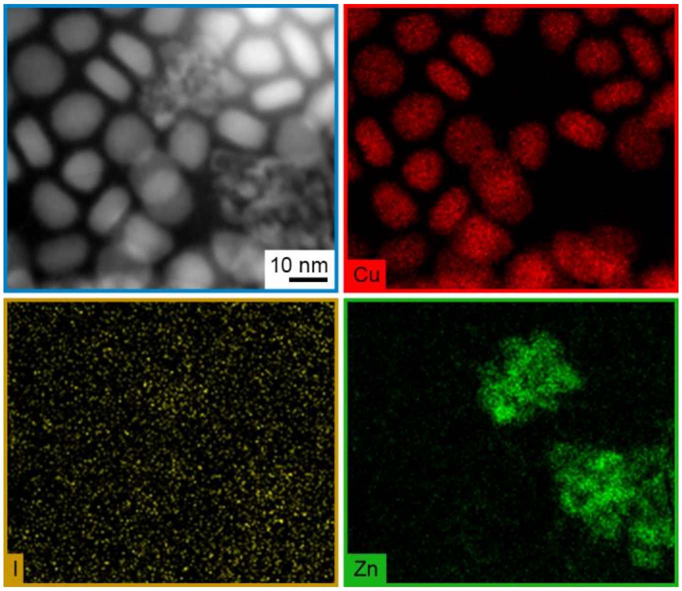

Figure S32. STEM–EDS mapping images of $Cu_{3-x}P$ nanocrystals oxidized with ZnI_2/TOP (Figure 5i). Mapping reveals localization of the Zn-Kα signal orthogonal to the Cu-Kα signal from the nanocrystals.

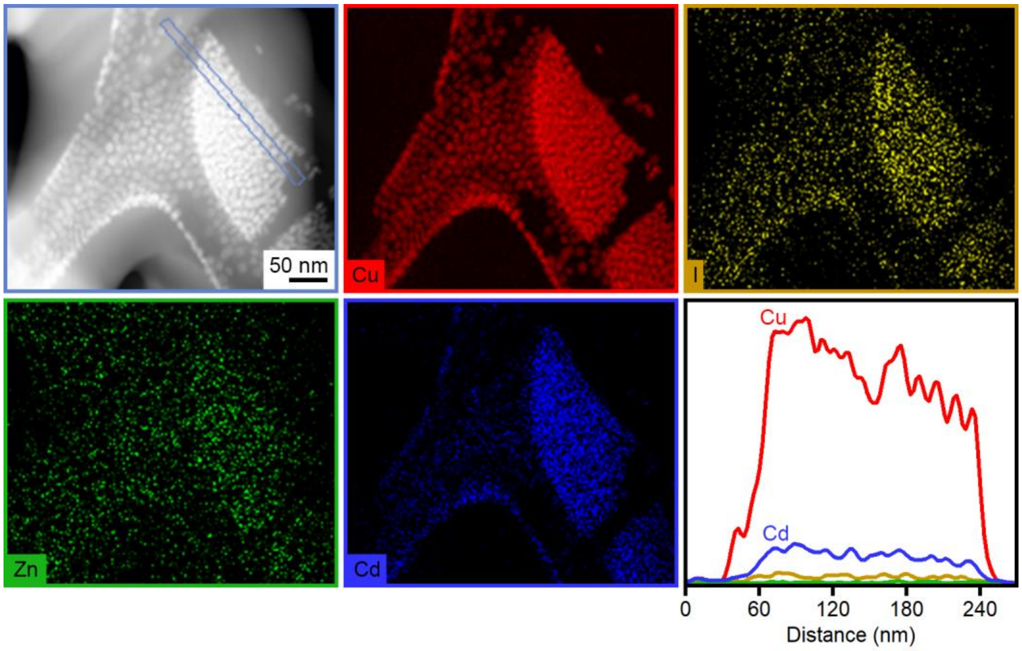

Figure S33. STEM–EDS mapping images and corresponding line-scan of $Cu_{3-x}P$ nanocrystals oxidized with CdI_2/TOP (Figure 5iii). Line-scan noise was reduced through binomial smoothing over a 5-nm range of pixels. Mapping reveals a co-localization of the Cd-Lα and I-Lα signals with the Cu-Kα signal from the nanocrystals.

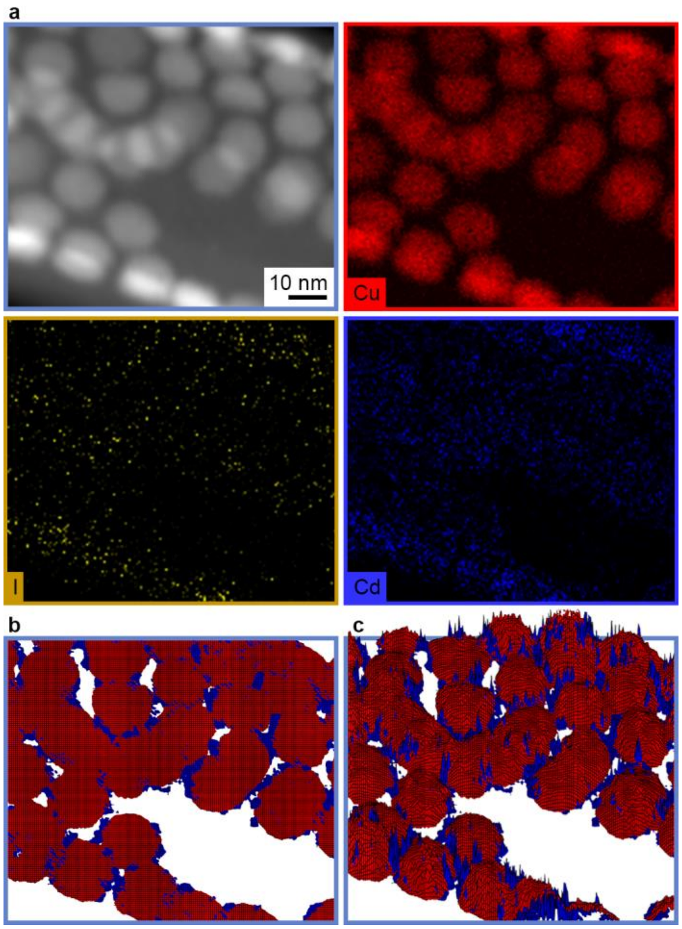

Figure S34. (a) STEM–EDS mapping images of $Cu_{3-x}P$ nanocrystals oxidized with CdI_2/TOP (Figure 5iii). **(b)** Top-down 2D projection and **(c)** 3D map with arbitrarily scaled Cu-Kα signal (red) and Cd-Lα signal (blue) after application of a pixel-wise adaptive low-pass Wiener filter using 5×5 pixel bins (514 pm pixel-length, 6.6 nm^2 area) and exclusion of signal below a noise z-limit.

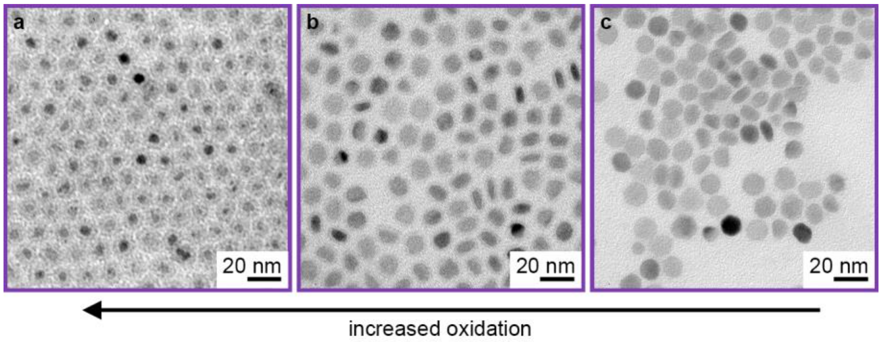

Figure S35. TEM images of $Cu_{3-x}P$ nanocrystals **(a)** stored aerobically for 4 months with 0 extra purifications (Synthesis 1). Heavy oxidation is evidenced by the appearance of low-contrast shells. **(b)** This oxidation is observed to a lesser extent when a similar batch of nanocrystals (Synthesis 8) are stored aerobically for only 42 h (0 extra purifications). **(c)** The same ensemble (Synthesis 8) with 3 extra washes shows no sign of oxidation after 42 h in air. All samples were stored in air as films on TEM grids.

Table S8. Comparison of $Cu_{3-x}P$ nanocrystal lateral size as determined by TEM imaging and calculated through Scherrer analysis of powder X-ray diffraction data.

Synthesis	Redox Treatment	TEM Lateral Size (nm)	Scherrer Lateral Size (nm)
1	As-synthesized	12.4 ± 0.7	11.9 ± 0.1
2	I_2	12.2 ± 0.6	11.0 ± 0.1
3	Cu(I)	12.9 ± 0.8	12.6 ± 0.6
4	ZnI_2/TOP	13.2 ± 0.7	12.2 ± 0.1
5	Li	12.1 ± 0.8	11.2 ± 0.1
6	As-synthesized	12.7 ± 0.8	11.6
6	ZnI_2/TOP then Cu(I)	12.3 ± 0.7	11.8
6	I_2 then Cu(I)	12.8 ± 0.7	12.0
7	As-synthesized	13.1 ± 0.6	11.4
7	ZnI_2/TOP	12.9 ± 0.7	11.3
7	$ZnCl_2$/TOP	12.9 ± 0.7	11.1
7	CdI_2/TOP	12.9 ± 0.7	11.3
7	$CdCl_2$/TOP	13.0 ± 0.7	11.1
7	$Zn(OAc)_2$/TOP	12.9 ± 0.7	10.5
8	As-synthesized	13.3 ± 0.9	12.2
NS1	As-synthesized	15 ± 1	14.3
NS1	Cu(I)	15 ± 1	14.3
NS1	ZnI_2/TOP	14 ± 1	14.0

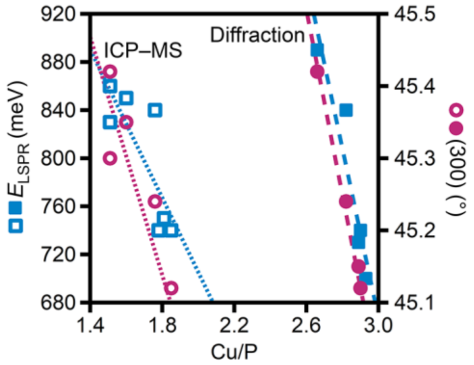

Figure S36. E_{LSPR} and (300) as a function of Cu/P, which was estimated from ICP–MS (open symbols) or diffraction data (closed symbols).

Table S9. Correlation between ΔCu and nanocrystal properties.

**Analysis Method for Cu/P**	**Cu/P**	**LSPR (meV)**	**(300)**	**Synthesis**	**Redox Treatment**
Diffraction	2.66	890	45.42	4	ZnI_2/TOP
	2.82	840	45.24	2	I_2
	2.90	740	45.12	1	None
	2.93	700	45.06	3	Cu^+
ICP–MS	1.51	860	45.42	6	ZnI_2/TOP
	1.51	830	45.30	7	CdI_2/TOP
	1.60	850	45.35	7	ZnI_2/TOP
	1.76	840	45.24	2	I_2
	1.78	740	45.09	6	None
	1.81	750	45.09	7	None
	1.85	740	45.12	1	None

Table S10. Chemicals

Chemical	Purity	Manufacturer
Chemicals used in $Cu_{3-x}P$ nanocrystal synthesis		
Copper(I) chloride (CuCl)	> 97 %	Strem Chemicals
Oleylamine (OAm)	> 98 %	Sigma Aldrich
Tris(diethylamino)phosphine ($P(NEt_2)_3$)	97 %	Alfa Aesar
Trioctylamine (TOA)	97 %	Frontier Scientific
Chemicals used for post-synthetic redox experiments		
n-Butyllithium (nBuLi)	-	Oakwood Chemical (1.6 M, hexane)
Cadmium chloride ($CdCl_2$)	> 99 %	Fisher Chemical (certified ACS)
Cadmium iodide (CdI_2)	98 %	Alfa Aesar
Cerium(IV) ammonium nitrate (CAN)	> 98.0 %	TCI Chemicals
Copper(II) triflate ($Cu(OTf)_2$)	98 %	Oakwood Chemical
Ferrocene	99 %	Alfa Aesar
Ferrocenium hexafluorophosphate ($FcPF_6$)	97 %	Sigma Aldrich
Lithium metal (Li^0)	> 99 %	Thermo Scientific Chemicals
Methanol (MeOH)	99.8 %	Sigma Aldrich (anh.)
Silver triflate (AgOTf)	99 %	Strem Chemicals
Tetrabutylammonium iodide (TBAI)	> 98 %	TCI Chemicals
Tetrakis(acetonitrile)copper(I) hexafluorophosphate ($[Cu(MeCN)_4]PF_6$)	> 98 %	Strem Chemicals
Trioctylphosphine (TOP)	> 97 %	Strem Chemicals
Zinc acetate dihydrate ($Zn(OAc)_2{\bullet}2H_2O$)	-	Fisher Chemical (cryst./certified)
Zinc chloride ($ZnCl_2$)	99 %	Sigma Aldrich (bioreagent)
Zinc iodide (ZnI_2)	> 98 %	Acros Organics
Other chemicals		
Benzene-d_6	99.5 %	Cambridge Isotope Laboratories
Copper metal	99 %	Alfa Aesar (−50+70 mesh, semisph.)
Copper(I) iodide (CuI)	98 %	Strem Chemicals
Nitric acid (HNO_3)	70 % bv	Fisher Chemical (certified ACS plus)
Nitric acid (HNO_3)	70 % bv	Fisher Chemical (TraceMetal)

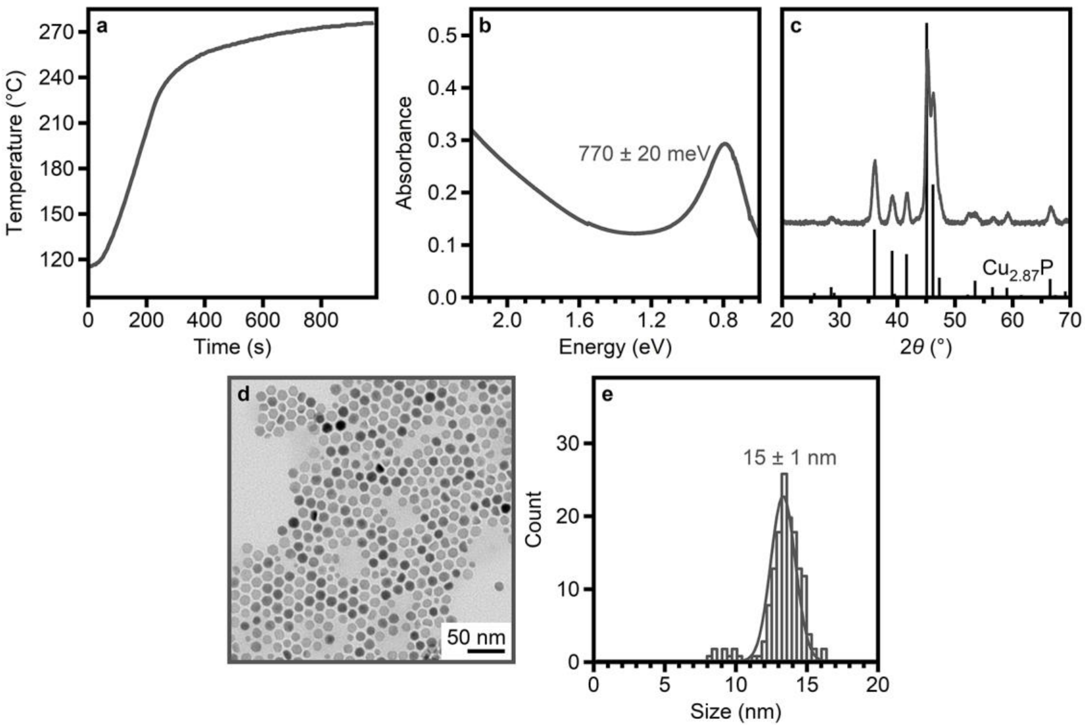

Figure S37. **(a)** Temperature-profile for the low-volume $Cu_{3-x}P$ nanocrystal heat-up synthesis (NS2, Table S1), which was heated with a contact voltage regulator. The maximum ramp-rate was $dT/dt \approx 35$ °C/min with $T_f = 276$ °C. **(b)** Absorption spectrum, **(c)** powder X-ray diffraction pattern (compared to that simulated for $P6_3cm$ $Cu_{2.87}P$, ref. 1), **(d)** TEM image and **(e)** statistical analysis ($n = 150$) of lateral platelet dimension of the resulting nanocrystals. Synthesis parameters are provided in Table S1.

References

1. Olofsson, O. The Crystal Structure of Cu_3P. *Acta Chem. Scand.* **1972,** *26*, 2777.
2. Jain, P. K.; Manthiram, K.; Engel, J. H.; White, S. L.; Faucheaux, J. A.; Alivisatos, A. P. Doped Nanocrystals as Plasmonic Probes of Redox Chemistry. *Angew. Chem. Int. Ed.* **2013,** *52*, 13671.
3. Hartstein, K. H.; Brozek, C. K.; Hinterding, S. O. M.; Gamelin, D. R. Copper-Coupled Electron Transfer in Colloidal Plasmonic Copper-Sulfide Nanocrystals Probed by in Situ Spectroelectrochemistry. *J. Am. Chem. Soc.* **2018,** *140*, 3434.
4. Elimelech, O.; Liu, J.; Plonka, A. M.; Frenkel, A. I.; Banin, U. Size Dependence of Doping by a Vacancy Formation Reaction in Copper Sulfide Nanocrystals. *Angew. Chem. Int. Ed.* **2017,** *56*, 10335.
5. Shan, Y.; Li, G.; Tian, G.; Han, J.; Wang, C.; Liu, S.; Du, H.; Yang, Y. Description of the Phase Transitions of Cuprous Iodide. *J. Alloys Compd.* **2009,** *477*, 403.
6. Fernandezgalan, R.; Manzano, B. R.; Otero, A.; Lanfranchi, M.; Pellinghelli, M. A. ^{19}Fand ^{31}P NMR Evidence for Silver Hexafluorophosphate Hydrolysis in Solution. New Palladium Difluorophosphate Complexes and X-Ray Structure Determination of [Pd(η^3-2-Me-C_3H_4)(PO_2F_2)(PCy_3)]. *Inorg. Chem.* **1994,** *33*, 2309.
7. Ono, H.; Ishimaru, S.; Ikeda, R.; Ishida, H. ^{1}H, ^{2}H, ^{19}F, ^{31}P, and ^{35}Cl NMR Studies on Molecular Motions in Ionic Plastic Phases of Pyrrolidinium Perchlorate and Hexafluorophosphate. *Bull. Chem. Soc. Jpn.* **1999,** *72*, 2049.
8. Hall, D. S.; Gauthier, R.; Eldesoky, A.; Murray, V. S.; Dahn, J. R. New Chemical Insights into the Beneficial Role of Al_2O_3 Cathode Coatings in Lithium-Ion Cells. *CS Appl. Mater. Interfaces* **2019,** *11*, 14095.
9. Xie, Y.; Riedinger, A.; Prato, M.; Casu, A.; Genovese, A.; Guardia, P.; Sottini, S.; Sangregorio, C.; Miszta, K.; Ghosh, S.; Pellegrino, T.; Manna, L. Copper Sulfide Nanocrystals with Tunable Composition by Reduction of Covellite Nanocrystals with Cu^+ Ions. *J. Am. Chem. Soc.* **2013,** *135*, 17630.
10. Melikyan, G. G.; Deravakian, A. Tetrahydrofuran as an One-Electron Donor: Highly Diastereoselective Coupling of Cobalt-Complexed Propargyl Alcohol. *J. Organomet. Chem.* **1997,** *544*, 143.
11. Lim, S. J.; Kim, W.; Shin, S. K. Surface-Dependent, Ligand-Mediated Photochemical Etching of CdSe Nanoplatelets. *J. Am. Chem. Soc.* **2012,** *134*, 7576.
12. Ievină, A.; Straumanis, M.; Karlsons, K. Praezisionsbestimmung von Gitterkonstanten hygroskopischer Verbindungen (LiCl, NaBr). *Z. Phys. Chem.* **1938,** *40B*, 146.
13. Luther, J. M.; Jain, P. K.; Ewers, T.; Alivisatos, A. P. Localized Surface Plasmon Resonances Arising from Free Carriers in Doped Quantum Dots. *Nat. Mater.* **2011,** *10*, 361.
14. Crovetto, A.; Unold, T.; Zakutayev, A. Is $Cu_{3-x}P$ a Semiconductor, a Metal, or a Semimetal? *Chem. Mater.* **2023,** *35*, 1259.
15. Agrawal, A.; Cho, S. H.; Zandi, O.; Ghosh, S.; Johns, R. W.; Milliron, D. J. Localized Surface Plasmon Resonance in Semiconductor Nanocrystals. *Chem. Rev.* **2018,** *118*, 3121.
16. Wolff, A.; Doert, T.; Hunger, J.; Kaiser, M.; Pallmann, J.; Reinhold, R.; Yogendra, S.; Giebeler, L.; Sichelschmidt, J.; Schnelle, W.; Whiteside, R.; Nimal Gunaratne, H. Q.; Nockemann, P.; Weigand, J. J.; Brunner, E.; Ruck, M. Low-Temperature Tailoring of Copper-Deficient $Cu_{3-x}P$ — Electric Properties, Phase Transitions, and Performance in Lithium-Ion Batteries. *Chem. Mater.* **2018,** *30*, 7111.

17. Wolff, A.; Pallmann, J.; Boucher, R.; Weiz, A.; Brunner, E.; Doert, T.; Ruck, M. Resource-Efficient High-Yield Ionothermal Synthesis of Microcrystalline $Cu_{3-x}P$. *Inorg. Chem.* **2016,** *55*, 8844.
